# Integrative Transcriptomic and Metabolomic Analyses of Time-of-Day Variation of Quality-Related Metabolites in Fresh Tea Leaves

**DOI:** 10.3390/plants15172558

**Published:** 2026-08-22

**Authors:** Liangjie Niu, Chunhui Wang, Jiayin Xie, Lin Cheng, Qiying Zhou, Wei Wang

**Affiliations:** 1 Dabie Mountain Laboratory, Henan Key Laboratory of Tea Plant Biology, Henan International Joint Laboratory of Tea-Oil Tree Biology and High-Value Utilization, College of Tea and Food Science, Xinyang Normal University, Xinyang 464000, China; xiejiayin@xynu.edu.cn (J.X.); lzc5569@xynu.edu.cn (L.C.); 2 College of Life Sciences, Henan Agricultural University, Zhengzhou 450046, China; 17732214584@stu.henau.edu.cn (C.W.); wangwei@henau.edu.cn (W.W.)

**Keywords:** leaf quality, metabolic dynamics, multi-omics, secondary metabolites, tea plant, time-of-day-dependent changes

## Abstract

Green tea quality is largely determined by the metabolite profile of fresh leaves. However, the time-of-day-dependent dynamics of these metabolites remain largely unknown in Xinyang Maojian (XYMJ), a premium Chinese green tea. In this study, we performed the first integrative transcriptomic and metabolomic analysis of *Camellia sinensis* cv. Xinyang 10 shoots (one bud and one leaf) sampled at 7:00, 13:00, and 18:00 under field conditions with light intensities of 19.2, 113, and 5.4 klx, respectively. We identified 525 differentially accumulated metabolites and 19,767 differentially expressed genes exhibiting distinct time-of-day-dependent patterns. Physiological measurements confirmed significant fluctuations in starch, soluble sugars, chlorophyll, relative water content, and polyphenols throughout the daytime. A key finding was a daytime carbon allocation trade-off: primary metabolism (starch biosynthesis, glycolysis, TCA cycle) peaked at 13:00, whereas secondary metabolism (flavonoids, theaflavins, phenolic acids, anthocyanins) dominated at 18:00, supported by strong negative correlations between primary and secondary metabolic modules. Chlorophyll and oligomeric catechins peaked at 7:00; theaflavins at 13:00; and starch, soluble sugars, theanine, and organic acids at 18:00. Light-responsive transcription factors (bZIP, NF-Y, HD-Zip, SPL, ARF, MADS) and other regulators (MYB, AP2/ERF, WRKY, NAC, GRAS, bHLH) exhibited time-specific expression, sequentially modulating flavonoid, caffeine, and theanine biosynthesis, along with specific gene modules including *SS3/SS4* (starch synthesis), *BAM3* (starch degradation), *FBA1* (carbon fixation), *CYP72A219* (terpenoid metabolism), and *L7A* (theaflavin biosynthesis). Evening-harvested leaves accumulated higher levels of theanine (umami) and soluble sugars (sweetness), whereas morning leaves were enriched in astringent catechins and flavonols. This multi-omics dissection of time-of-day-dependent metabolism in XYMJ tea provides a scientific basis for time-of-day harvesting strategies and graded processing of tea products.

## 1. Introduction

Tea plant (*Camellia sinensis*) is an economically important woody crop globally. Tea is the world’s oldest and most widely consumed non-alcoholic beverage, second only to drinking water, with an estimated 3 billion consumers across more than 160 countries [1]. The economic and sensory values of tea are largely determined by the secondary metabolites present in its fresh shoots, such as catechins, theanine, and caffeine, which are key contributors to tea flavor and quality [2]. Specifically, catechins are the main contributor to astringency [3]; theanine confers fresh, mellow, and umami flavors to tea infusion, whereas caffeine serves as the primary source of bitterness [4]. Synergistic interactions and balanced concentration ratios among catechins, theanine, caffeine, and other metabolites ultimately shape the characteristic taste profile of finished tea [5]. Moreover, these secondary metabolites offer multiple health benefits, including antioxidant, anticancer, cholesterol-lowering and antihypertensive effects [6].

Tea plants have been cultivated in China for over 2000 years, with systematic plantations established as early as the Jin Dynasty (265–420 AD). Diversified cultivars and region-specific processing methods enable the production of hundreds of tea types across China [7]. Among the tea-growing regions, Xinyang City, Henan Province, has a cultivation history of over 1000 years and represents the northernmost major green tea-producing area in China. Xinyang Maojian (XYMJ) is one of China’s top ten famous green teas [8] and is typically produced from spring tender shoots with one bud and one leaf from small-leaf tea cultivars (*C. sinensis* var. *sinensis*) [9]. Its core producing area lies in southern Xinyang, located in the middle and upper reaches of the Huaihe River, featuring a transitional climate between subtropical and temperate zones, with an average annual rainfall of 1100 mm, annual sunshine duration of 2200 h, and a mean annual temperature of approximately 15 °C—lower than that of other green tea-producing regions [8]. Local tea plants are mainly cultivated at altitudes of 300–800 m and latitudes of 31–33° N [10]. These unique geographical and climatic conditions, combined with distinctive processing techniques, favor the growth of high-quality tea leaves, endowing XYMJ with superior quality, distinctive flavor, and rich nutritional value.

Tea fresh leaves accumulate a wide range of metabolites, including amino acids, flavonoids, phenolic acids, volatiles, and their derivatives [11,12]. The initial metabolic profile of fresh leaves, which is primarily governed by cultivar genotype, cultivation management, and growing environments, is a core determinant of tea quality, flavor and bioactive health-promoting properties after processing [10,13,14]. Moreover, the contents of catechins, phenolic acids, amino acids, flavonoids, and flavone glycosides vary considerably among tea cultivars [15], and each phylogenetic group contains characteristic signature metabolites [4]. The biosynthesis of catechins, caffeine, and theanine in *C. sinensis* exhibits significant seasonal variations [16], and the aroma of green tea is jointly shaped by environmental conditions and leaf harvesting time [17,18].

Among these factors, light acts as the core environmental signal governing photosynthesis and the biosynthesis of quality-related metabolites [19,20]. Daytime changes in light intensity modulate dynamic photosynthetic activity and subsequent secondary metabolite accumulation in tea plants, exerting a markedly stronger regulatory effect than other environmental variables over multi-seasonal time scales [16]. Under high irradiation, excess photosynthates are rapidly converted into other metabolites; meanwhile, dynamic histone modifications markedly upregulate the expression of enzyme genes involved in secondary metabolic pathways as part of the plant’s adaptive response [21]. Plant growth and metabolite levels also display clear time-of-day-associated variation. Leaf starch, in particular, undergoes marked diurnal changes: it is synthesized and accumulates under light, peaks at the end of the light period, and is degraded during the night [22,23]. Starch metabolism significantly modulates the accumulation of secondary metabolites (polyphenols, caffeine and theanine) in tea buds and young leaves. Glycolysis-derived intermediates from starch degradation, such as pyruvate and glutamine, serve as precursors for theanine synthesis; phenylalanine contributes to tea polyphenol and flavonoid biosynthesis, and purines act as caffeine precursors [24].

The picking time of fresh tea leaves directly determines the yield and quality of finished tea products, as time-of-day-dependent fluctuations in light, temperature, humidity, and CO_2_ profoundly influence the accumulation of flavor-related metabolites [24,25]; inappropriate picking timing compromises this accumulation and reduces leaf quality. Therefore, determining the optimal intra-day picking time is of great practical value. However, research on XYMJ tea has focused predominantly on processing optimization, sensory evaluation, and long-term seasonal adaptation, and no systematic metabolomic or transcriptomic study has been conducted at the intra-day scale. In particular, the correlation between starch and key secondary metabolite accumulation at different intraday time points remains unclear.

In this study, we performed integrative transcriptomic and metabolomic analyses to explore time-of-day-dependent changes in gene expression and metabolite accumulation in buds and young leaves of *C. sinensis* cv. Xinyang 10 (XY10). We aimed to elucidate how intra-day picking time affects the metabolic profiles of fresh tea leaves and to reveal the molecular and physiological patterns associated with leaf quality formation, thereby providing theoretical and technical support for scientific plucking and refined tea processing.

## 2. Result

### 2.1. Variation in Quality Indexes Across Different Samples

**Figure 1:**
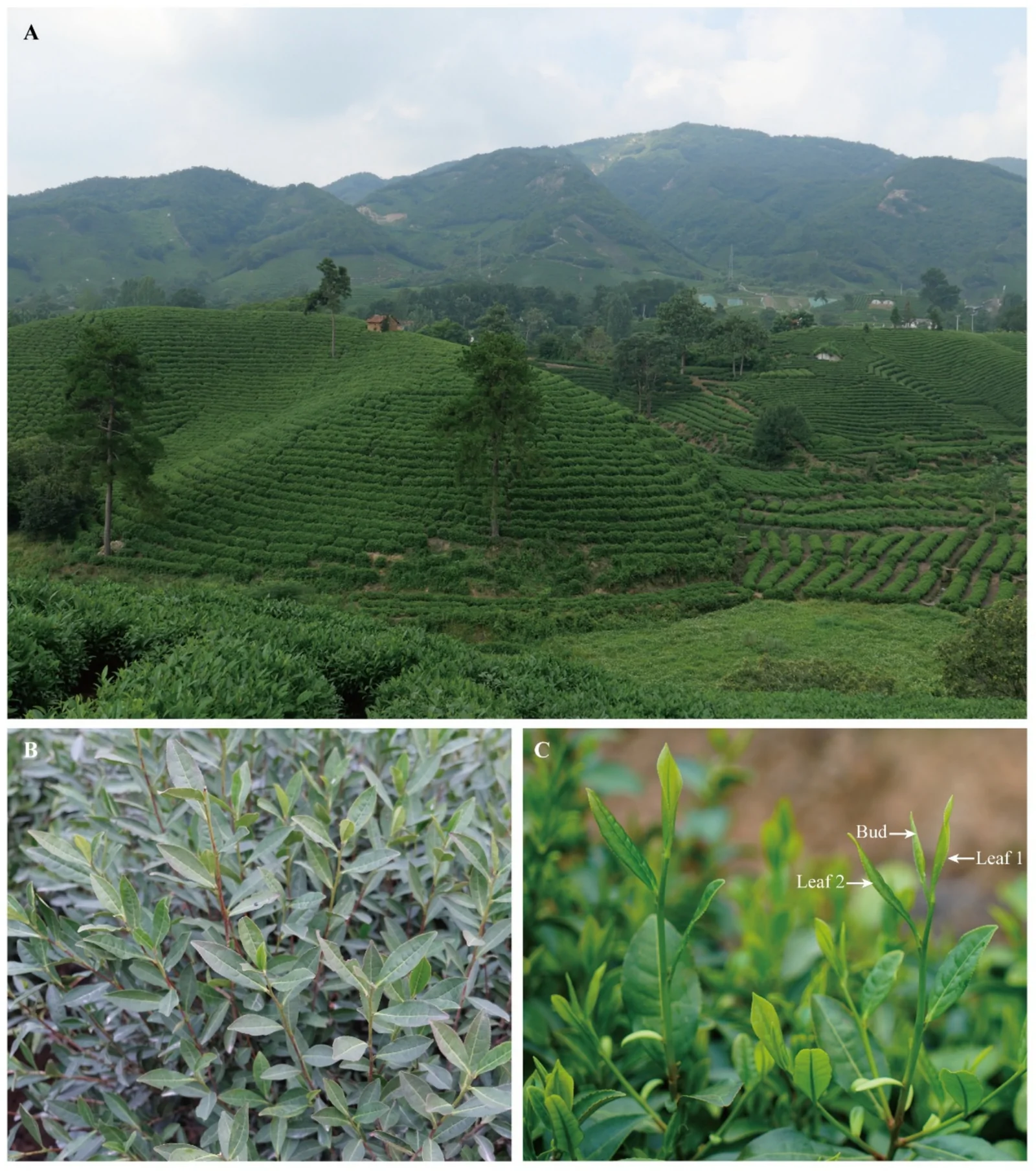
Tea plantation, morphology, and sampling of XY10 tea plant. (**A**) The tea plantation at Bailong Pool, Shihe District, Xinyang City, Henan Province, China. (**B**) Overwintering seedlings of XY10, photographed in February 2025. (**C**) New spring shoots of XY10, photographed during the Grain Rain period in April 2025. For physiological and metabolomic analyses, tea shoots (one bud and one leaf) of XY10 were randomly sampled at 7:00, 13:00 and 18:00, respectively. The environmental parameters at the three sampling time points were as follows: 19.2 klx, 17 °C, 88% relative humidity (RH); 113 klx, 29 °C, 52% RH; and 5.4 klx, 24 °C, 58% RH. Sunrise and sunset on the sampling day were recorded at 05:50 and 18:55, respectively.

The experimental tea plantation is situated at Bailong Pool (near 32° N) in the core XYMJ tea-growing region in Xinyang City, China (Figure 1A). Significant time-of-day-dependent variations in major environmental factors, including light intensity, air temperature, and relative humidity, were recorded at the three sampling time points (7:00, 13:00, and 18:00) within a single sunny sampling day. These variations inevitably affected photosynthesis and the accumulation of endogenous compounds in tea plants. From the overwintered XY10 tea plant population (Figure 1B), fresh spring shoots (one bud and one or two leaves) were randomly collected after sprouting (Figure 1C).

**Figure 2:**
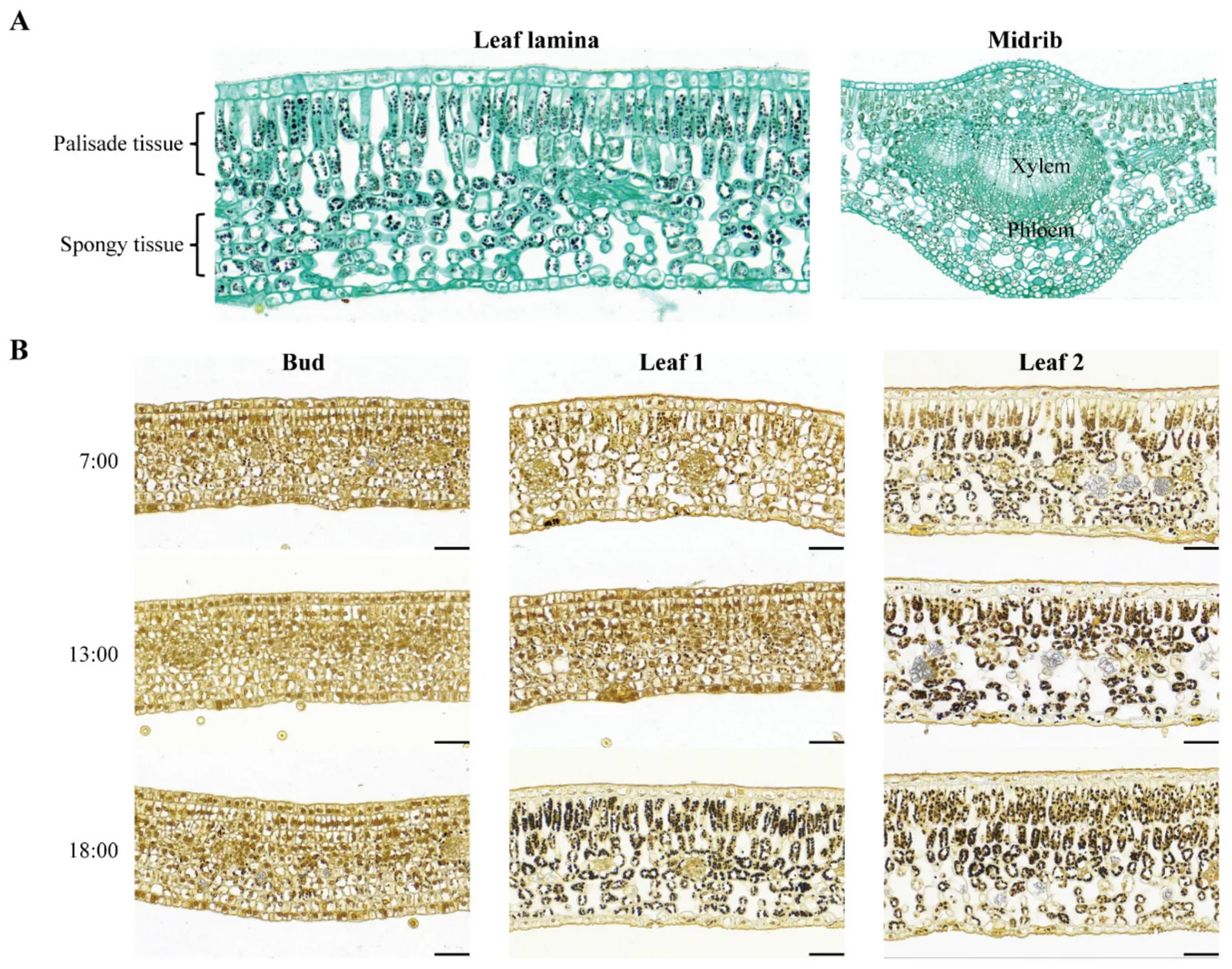
Microstructure and daytime variation of starch accumulation in fresh tea leaves. Sections were prepared from separate bud, first-leaf, and second-leaf tissues (distinct from the one-bud-one-leaf samples used for physiological and metabolomic analyses), stained with safranin-fast green (**A**) or iodine (**B**), and observed under light microscopy. Starch granules in mesophyll cells were stained dark blue (indicated by arrows). Scale bar: 50 μm.

The anatomical traits and photosynthate accumulation of fresh tea leaves were closely associated with leaf development and sampling time (Figure 2). As shown in Figure 2A, the second leaf possessed well-differentiated palisade and spongy tissues, characterized by regularly arranged cells, abundant intercellular spaces, and fully developed midrib vascular bundles with distinct xylem and phloem differentiation. In contrast, the bud and the first leaf exhibited underdeveloped mesophyll tissues, obscure boundaries between palisade and spongy tissues, and compact cell arrangement (Figure 2B). Additionally, the thickness of the epidermal cuticle increased with leaf maturation.

In iodine-stained leaf cross sections, dark-blue starch granules primarily accumulated in palisade and spongy tissues, and starch abundance increased markedly from morning to afternoon across all samples (Figure 2). Starch levels remained low at 7:00, accumulated massively by 13:00, and peaked at 18:00. Notably, the second leaf accumulated more starch than the bud and the first leaf (Figure 2B). Overall, starch accumulation differed significantly with both sampling time and leaf maturity. A similar starch accumulation pattern was observed in the main veins of all three sample types (Figure S1).

**Figure 3:**
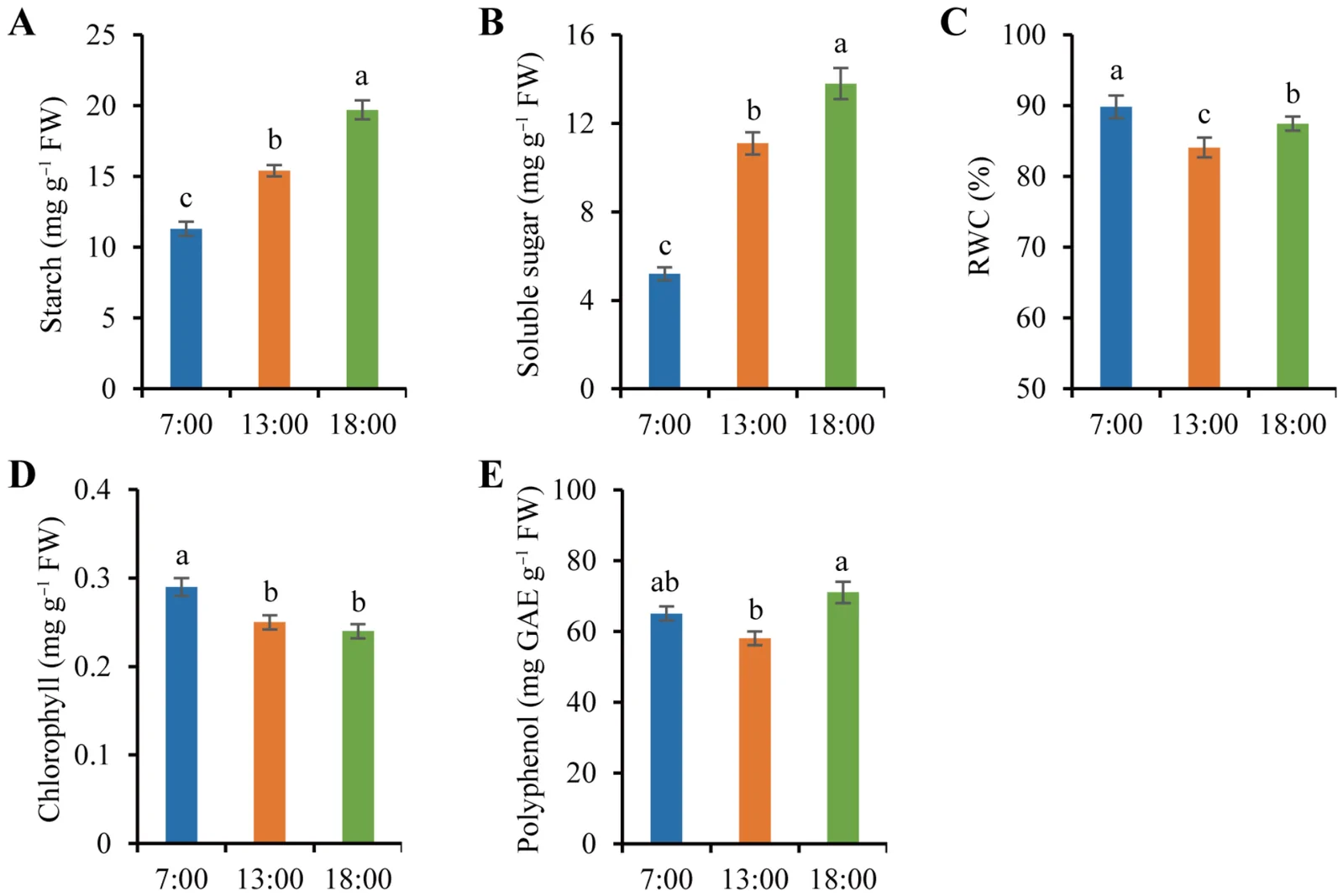
Dynamic changes in tea shoots at different picking times. (**A**) Starch content. (**B**) Soluble sugar content. (**C**) RWC. (**D**) Chlorophyll content. (**E**) Polyphenol content. Data are presented as mean ± SD (*n* = 3). The different letters above the bars indicate statistically significant differences (*p* < 0.05) according to one-way ANOVA with Tukey’s test.

To characterize time-of-day-dependent physiological variations in tea shoots, we analyzed five key physiological parameters in samples collected at three time points (Figure 3). Both starch and soluble sugars accumulated progressively, peaking at 18:00, reflecting sustained photosynthetic carbon assimilation throughout the day. Relative water content (RWC) was highest at 7:00 (~90%), declined to its minimum at 13:00 (~85%) due to midday transpiration, and partially recovered by 18:00 (~88%). Chlorophyll content was highest at 7:00 and decreased gradually to its lowest level by 18:00. Polyphenol content followed a V-shaped pattern, with the lowest values at 13:00 and the highest at 18:00. These changes represent the inherent physiological responses of tea plants to time-of-day-dependent fluctuations in light intensity, air temperature, and relative humidity. To further explore the metabolic differences among the three time points, we subsequently performed an untargeted metabolomic analysis.

### 2.2. Changes in Metabolic Profiles of XY10 Tissues at Different Sampling Times

#### 2.2.1. Overview of Metabolome Changes

**Figure 4:**
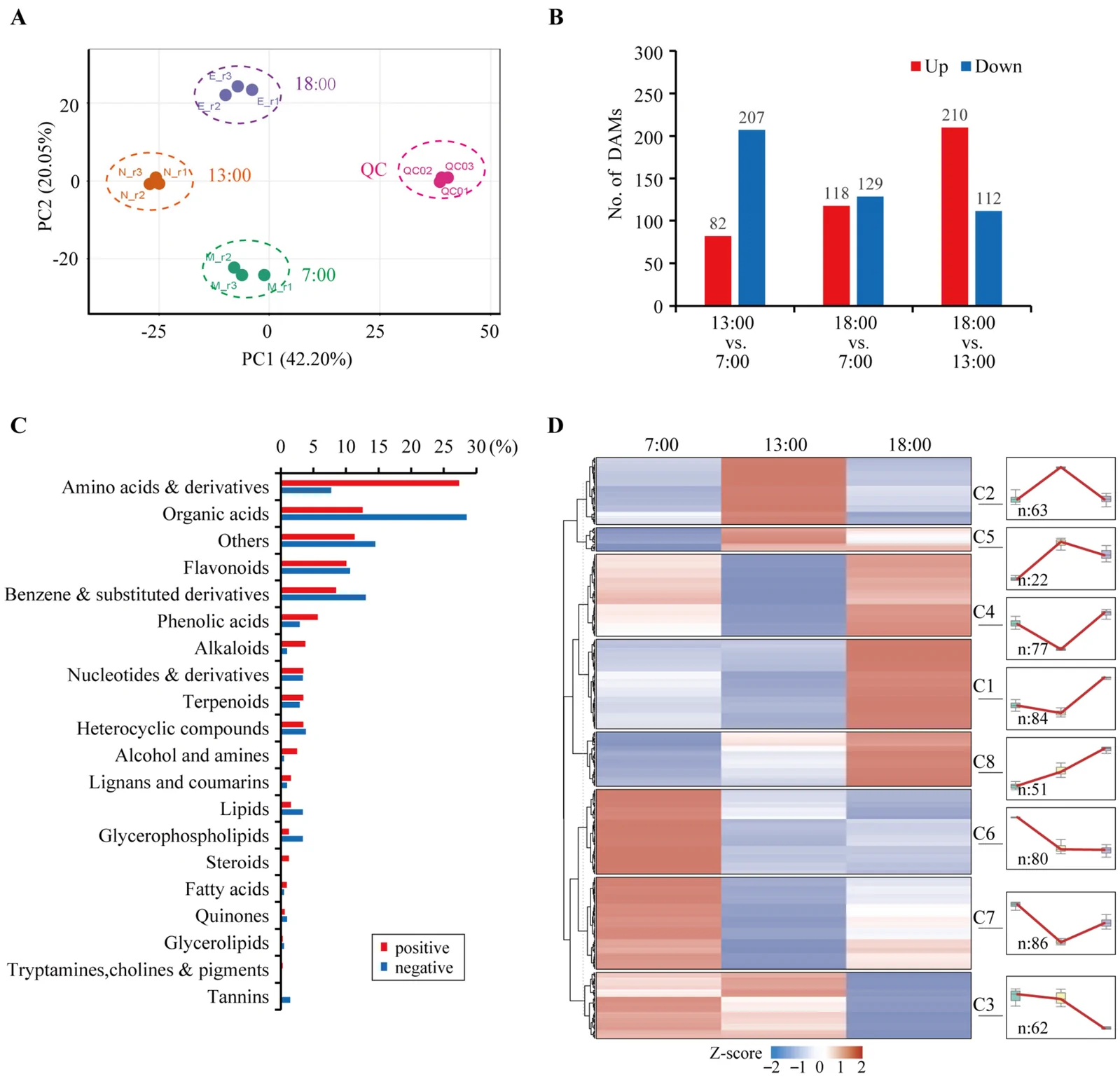
Overall results of metabolomic data of tea tissue samples collected at 7:00, 13:00 and 18:00, respectively. (**A**) PCA of metabolomic data obtained from both positive ion and negative ion modes. PC1 (first principal component, 42.20% of total variance); PC2 (second principal component, 20.05% of total variance). Quality control (QC) samples clustered tightly, and samples from the three time points formed well-separated clusters, indicating high data quality and reproducibility. (**B**) Number of DAMs. Screening criteria: VIP > 1, fold change ≥ 2. After removing DAMs shared across the three comparisons, 525 unique DAMs remained (see Table S1). (**C**) The proportional distribution of different categories of DAMs. Data were ordered by abundance in the positive ion mode. (**D**) HCA of DAMs.

Unsupervised principal component analysis (PCA) was performed on metabolomic data of XY10 samples harvested at three time points (7:00, 13:00, and 18:00). The first two principal components (PC1 and PC2) explained 42.20% and 20.05% of the variance, respectively. A prominent time-of-day-associated shift in the metabolome was observed, particularly between 18:00 and 7:00, highlighting sampling time as a primary source of variation (Figure 4A). Such pronounced metabolic separation across sampling times may reflect the combined influence of daytime light and temperature fluctuations, as reported for photosynthetic plant species [19,21]. Specifically, the metabolic profiles at 7:00 largely reflect overnight metabolic activities, whereas those at 18:00 are predominantly derived from daytime photosynthesis and coupled secondary metabolic pathways.

A total of 1483 metabolites were detected in all samples using both positive and negative ion modes. Among them, 525 differentially accumulated metabolites (DAMs) were screened via pairwise comparisons using variable importance in projection (VIP) > 1 and fold change ≥ 2, including both up- and down-accumulated metabolites (Figure 4B, Table S1). These DAMs mainly included amino acids and their derivatives, organic acids, flavonoids, benzenoids and their derivatives, and phenolic acids (Figure 4C). Notably, amino acids and derivatives accounted for 26% in positive ion mode but only 10% in negative ion mode. Organic acids were notably more abundant (28%) in the negative ion mode. Most other metabolites were more abundant in positive ion mode.

Hierarchical clustering analysis (HCA) of DAMs identified eight time-of-day-dependent accumulation clusters (C1–C8) with divergent temporal profiles at 7:00, 13:00, and 18:00 (Figure 4D). C2 and C5 peaked at 13:00, while C1, C4, and C8 attained maximal abundance at 18:00. C3, C6 and C7 accumulated to high levels at 7:00 and declined gradually over time; in particular, C7 displayed a V-shaped profile, with its minimum at 13:00. Collectively, these findings demonstrate that metabolic processes in tea shoots are dynamically modulated at different time points over the course of the day.

#### 2.2.2. Specific Changes in Key Metabolites Related to Tea Leaf Quality

To better characterize chemical discrepancies across three harvesting time points, we screened 54 key quality-related compounds (Table 1) from 525 DAMs (Table S1), to highlight their contributions to tea quality and their time-of-day-dependent shifts in relative abundance. These compounds, including alcohols and amines, amino acid derivatives, benzene derivatives, flavonoids, organic acids, phenolic acids, and tannins, are closely correlated with multiple tea sensory attributes: infusion color, aroma, freshness, sweetness, astringency, and mellowness. Significant time-of-day-dependent differences were observed in the relative contents of most metabolites across the three sampling time points (*p* < 0.05). Most amino acids, aroma precursors, and sweetness- and mellowness-related compounds exhibited maximum accumulation at 18:00. Certain flavonoids, phenolic acids, and astringent substances reached their highest levels at 7:00, whereas theaflavins and polymeric flavanols were markedly accumulated at 13:00. Notably, metabolites belonging to the same class frequently displayed divergent temporal profiles.

Theanine, the most abundant umami amino acid, increased from 7:00 to 18:00, along with pyroglutamic acid and lysine, whereas reduced glutathione peaked at 7:00 and proline at 13:00. Organic acids usually were high at 7:00, lowest at 13:00, and recovered by 18:00. Most benzene derivatives were lower at 13:00 than at 7:00, except procyanidin Trimer-Vanillyl 1 (peaked at 13:00). Flavonoids displayed diverse time-of-day-dependent accumulation patterns. GC-(4α→8)-GC-(4α→8)-GC and [GC-(4α→8)]_2_-catechin peaked at 13:00, whereas GC-(4α→8)-EGC remained high at 7:00 and 13:00 before declining by 18:00. Rutin, hyperoside, myricitrin, quercetin and quercetagetin steadily decreased from 7:00 onward. In contrast, mangiferin, sakuranetin, naringenin-7-O-D-glucuronide, and maclurin increased substantially by 18:00, while cyanidin derivatives and morin showed little time-of-day-dependent variation. Five phenolic acids peaked at 7:00 and then decreased markedly by 13:00, whereas 1,3-dicaffeoylquinic acid peaked at 13:0 and declined gradually by 18:00. Three theaflavin-derived tannins reached the lowest levels at 7:00, peaked at 13:00, and decreased gradually by 18:00. Polysaccharides (e.g., trehalose, cellotetraose) accumulated to higher levels at 13:00, whereas soluble sugars like fructose peaked at 18:00. Collectively, these results reveal obvious time-specific metabolic reprogramming in tea leaves.

#### 2.2.3. Kyoto Encyclopedia of Genes and Genomes (KEGG) Pathway and Metabolite Correlation Analyses

**Figure 5:**
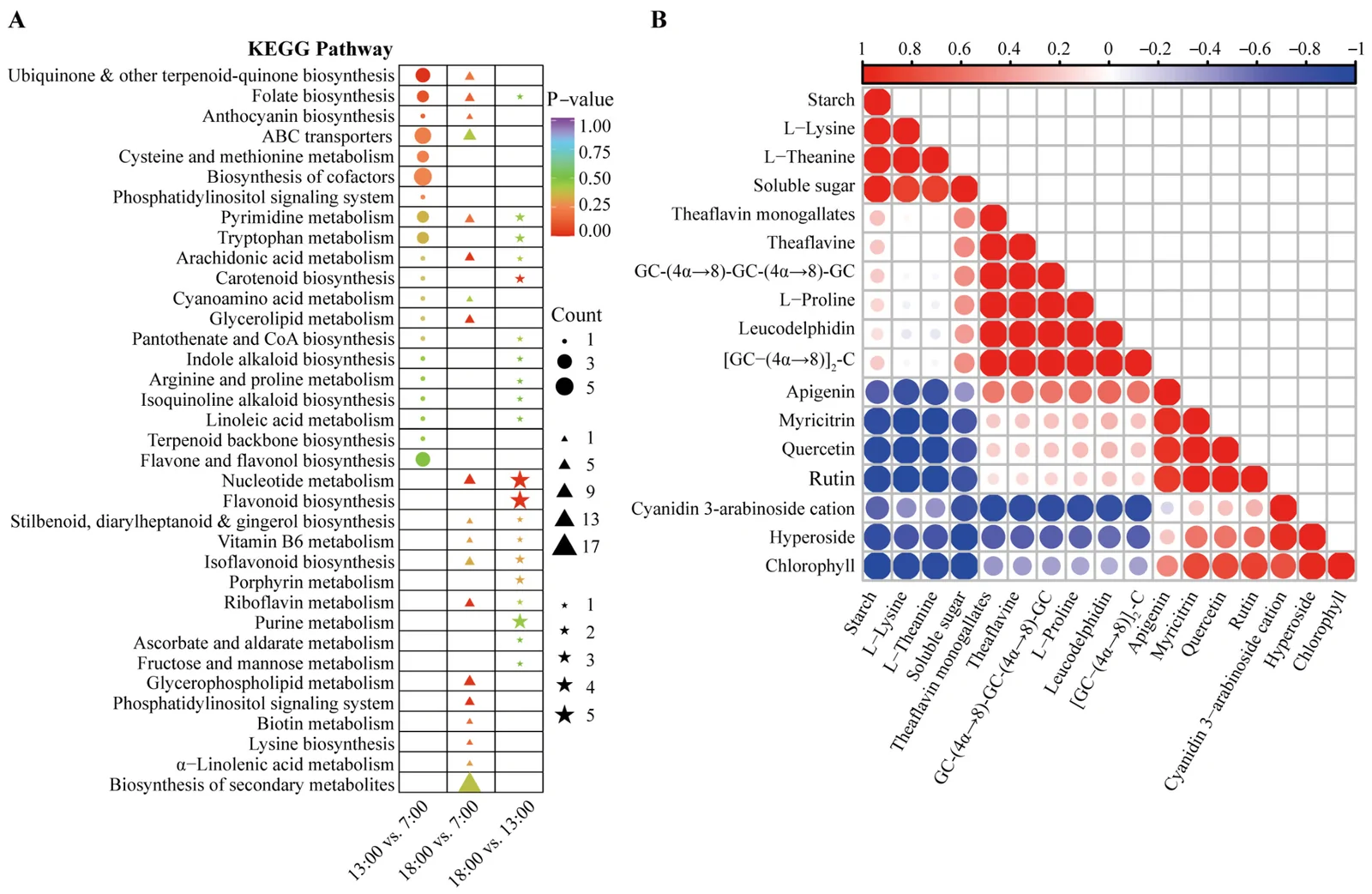
KEGG enrichment of DAMs and correlation analysis of key DAMs accumulation in tea tissue samples collected at 7:00, 13:00 and 18:00, respectively. (**A**) KEGG pathway enrichment analysis of DAMs responding to time-of-day-dependent variation. (**B**) Correlation heatmap based on Pearson correlation. Numerical values for all pairwise correlations are listed in Table S2.

KEGG pathway enrichment analysis (Figure 5A) revealed significant enrichment of multiple pathways in the 13:00 vs. 7:00 comparison, including ubiquinone and other terpenoid-quinone biosynthesis, folate biosynthesis, ABC transporters, cysteine and methionine metabolism, cofactor biosynthesis, and pyrimidine plus tryptophan metabolism. Among them, ABC transporters coordinately mediate intracellular redistribution of metabolites. Enhanced photosynthesis drives synchronous activation of primary metabolism. In the 18:00 vs. 13:00 comparison, metabolic variation was dominated by flavonoid biosynthesis and nucleotide metabolism, together with elevated biosynthesis of carotenoids, isoflavonoids and porphyrins. DAMs from the 18:00 vs. 7:00 comparison partially overlapped with those of 18:00 vs. 13:00, while more secondary metabolic pathways were substantially enriched, such as glycerolipid, glycerophospholipid, riboflavin, biotin (vitamin B7), and lysine metabolism.

The heatmap (Figure 5B) revealed a strong negative correlation between primary metabolites (amino acids and carbohydrates) and secondary metabolites (polyphenols, flavonoids and pigments) in tea leaves over the course of the day, suggesting competitive carbon allocation between primary and secondary metabolism. Starch, soluble sugars and theanine were positively correlated with one another; the same positive association existed among theaflavins, theaflavin monogallates and catechin oligomers. Catechin oligomers ([GC-(4α→8)-GC-(4α→8)-GC, [GC-(4α→8)]_2_-C), flavonols (apigenin, myricitrin, quercetin, rutin, hyperoside), anthocyanins (cyanidin 3-arabinoside cation, leucodelphidin), and other pigments (theaflavins, chlorophyll) were negatively correlated with primary metabolites. Collectively, this antagonistic metabolite accumulation highlights clear time-of-day-dependent metabolic shifts and carbon allocation trade-offs between primary and secondary metabolism in tea shoots, laying a theoretical foundation for optimizing harvest timing and guiding hierarchical tea processing.

### 2.3. Analysis of Differentially Expressed Genes (DEGs) in Tea Shoots at Different Picking Times

Transcriptome profiling was performed on XY10 tea shoots across three sampling time points to screen core DEGs, thereby uncovering the molecular basis underlying time-of-day-dependent metabolomic fluctuations across samples. PCA based on transcriptome sequencing (RNA-seq) datasets from samples harvested at 7:00, 13:00 and 18:00 showed obvious time-dependent separation (Figure S2A), consistent with the PCA results of metabolome data (Figure 4A). Pairwise comparisons via DESeq2 identified 19,767 DEGs comprising both up- and down-regulated transcripts (Figure S2; Table S3). Remarkably, the number of DEGs identified at distinct time points (Figure S2B) was quantitatively comparable to that of DAMs identified above (Figure 4B).

These DEGs were involved in primary and secondary metabolic pathways (Figure S2D). The 13:00 vs. 7:00 comparison enriched the most extensive pathways, including photosynthesis, protein biosynthesis, sphingolipid metabolism, fatty acid metabolism, and ABC transporters (consistent with the KEGG enrichment results of DAMs in Figure 5A), which reveals extensive transcriptional reprogramming from morning to noon. The cytosolic DNA-sensing, ubiquitin-mediated proteolysis, and chloroalkane/chloroalkene degradation pathways were significantly enriched, indicating activation of stress defense and detoxification cascades under high irradiance and elevated temperature at noon. The 18:00 vs. 7:00 comparison was predominantly enriched in plant circadian rhythm pathway, glutathione metabolism, and secondary metabolite biosynthesis pathways, with the latter showing the highest enrichment significance and the most enriched DEGs. The 18:00 vs. 13:00 comparison showed fewer enriched pathways, mainly covering photosynthesis antenna proteins, endoplasmic reticulum protein processing, glutathione metabolism, and calcium signaling. Photosynthesis pathways were consistently enriched across all comparisons, highlighting their pivotal functions in tea plants. These results indicate highly coordinated metabolic regulation in tea leaves across the three daytime sampling points, covering photosynthesis, carbon partitioning, secondary metabolism, photoprotection, and stress defense.

We further compared enrichment ratios and biological functions of core transcription factors (TFs) across samples collected at three time points and identified MYB, AP2/ERF, WRKY, NAC, and GRAS as the dominant TF families (Table 2 and Table S4). Notably, highly enriched MYB at 13:00 vs. 7:00 regulates flavonoid and polyphenol biosynthesis, consistent with their time-of-day-dependent fluctuation (Table 1). AP2/ERF increased at 18:00 vs. 13:00, involved in caffeine biosynthesis and stress defense. WRKY, NAC, and GRAS co-regulate flavonoid and theanine biosynthesis, matching their massive accumulation at 18:00 (Table 1). Besides, bHLH forms MBW complexes with MYB to co-regulate flavonoid metabolism. Additional TFs (Zinc-finger, bZIP, TCP, etc.) regulate the time-specific accumulation of quality-related metabolites via light, hormone and epigenetic regulation. Specifically, a cohort of TFs functionally associated with light and photoperiod signaling was also enriched (Table 2). Among them, bZIP and NF-Y act as core light-responsive factors to mediate flavonoid and caffeine biosynthesis, while Homeobox/HD-Zip and SPL showed time-of-day-dependent expression patterns that correlated with tea growth, development and flavonoid metabolism. Likewise, MYB, ARF and MADS exhibited similar temporal correlations with the accumulation of theanine, flavonoids and other secondary metabolites. These light/photoperiod-responsive TFs displayed divergent enrichment at 7:00, 13:00 and 18:00, suggesting a potential transcriptional network through which tea plants respond to daytime light variations that correlate with fluctuations of key metabolites.

### 2.4. Integrative Transcriptomic and Metabolomic Analysis

**Figure 6:**
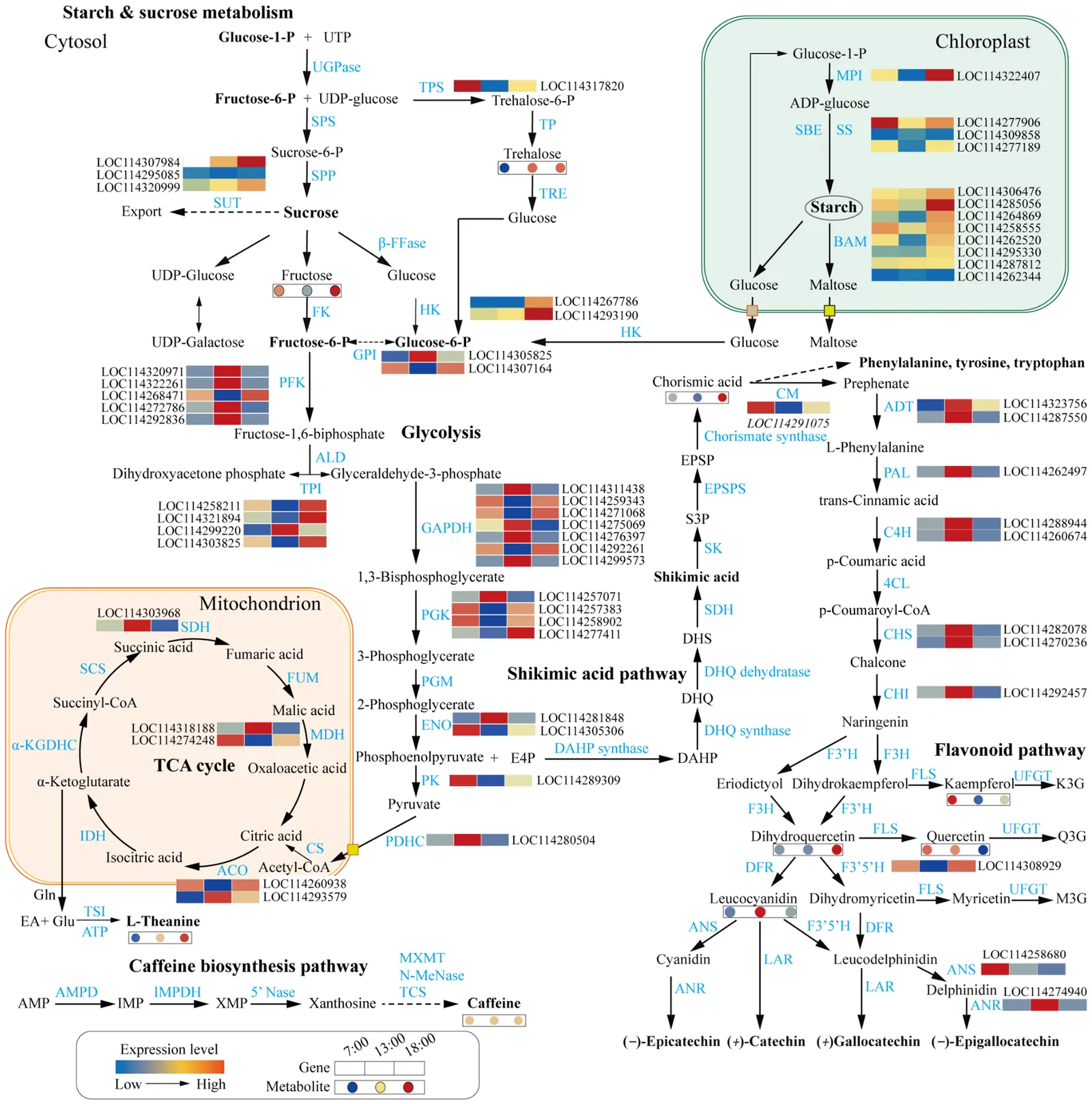
Integrative transcriptomic and metabolomic analysis of time-of-day-dependent expression of DEGs and DAMs related to primary and secondary metabolism in tea leaves. Abbreviations: 4CL, 4-coumarate CoA ligase; 5′Nase, 5′-nucleotidase; ACO, aconitate hydratase; ADT, arogenate dehydratase; ALD, aldolase; AMPD, AMP deaminase; ANR, anthocyanidin reductase; ANS, anthocyanidin synthase; BAM, β-amylase; C4H, cinnamate 4-hydroxylase; CHI, chalcone isomerase; CHS, chalcone synthase; CM, chorismate mutase; CS, ATP-citrate synthase; DAHP, 3-deoxy-D-arabino-heptulosonate 7-phosphate; DAHP synthase, 3-deoxy-D-arabino-heptulosonate 7-phosphate synthase; DFR, dihydroflavonol 4-reductase; DHQ, 3-dehydroquinate; DHQ dehydratase, 3-dehydroquinate dehydratase; DHQ synthase, 3-dehydroquinate synthase; DHS, 3-dehydroshikimate; E4P, D-erythrose-4-phosphate; ENO, enolase; EPSP, 5-enolpyruvylshikimate-3-phosphate; EPSPS, 5-enolpyruvylshikimate-3-phosphate synthase; F3′H, flavonoid 3′-hydroxylase; F3H, flavanone 3-hydroxylase; F3′5′H, flavonoid 3′,5′-hydroxylase; FK, fructokinase; FLS, flavonol synthase; FUM, fumarase; GAPDH, glyceraldehyde-3-phosphate dehydrogenase; GPI, Glucose-6-phosphate isomerase; HK, hexokinase; IDH, isocitrate dehydrogenase; IMPDH, IMP dehydrogenase; LAR, leucoanthocyanidin reductase; MDH, malate dehydrogenase; MPI, Mannose-6-phosphate isomerase; MXMT, Methylxanthine methyltransferase; N-MeNase, N-methyl nucleosidase; PAL, phenylalanine ammonia-lyase; PDHC, pyruvate dehydrogenase complex; PGK, phosphoglycerate kinase; PGM, Phosphoglyceromutase; PK, pyruvate kinase; PFK, phosphofructokinase; S3P, shikimate-3-phosphate; SBE, starch-branching enzyme; SCS, succinyl-CoA synthetase; SDH, Shikimate dehydrogenase; SK, shikimate kinase; SS, starch synthase; SPS, sucrose-phosphatase synthase; SUT, sucrose transporter; TCS, tea caffeine synthase; TP, trehalose phosphate; TPI, triose-phosphate isomerase; TPS, trehalose-6-phosphate synthase; TRE, trehalase; UFGT, UDP-glucose flavonoid 3-O-glucosyltransferase; UGPase, UDP-glucose pyrophosphorylase; α-KGDHC, α-ketoglutarate dehydrogenase complex; β-FFase, β-fructofuranosidase.

Transcriptome–metabolome integration further revealed time-of-day-dependent expression patterns of key genes involved in primary and secondary metabolism in tea shoots (Figure 6). Starch synthase genes (*SS3*, *SS4*) reached expression maxima at 13:00, whereas several β-amylase (*BAM*) and mannose-6-phosphate isomerase 1 (*MPI*) genes showed higher expression at 7:00 and 18:00, indicating starch biosynthesis predominated during daytime and catabolism proceeded under dusk conditions. Sucrose transporter (*SUT2*, *SUC4*), trehalose-6-phosphate synthase (*TPS1*), and hexokinase-3 (*HK*) genes also peaked at 13:00 to facilitate sucrose translocation, trehalose enrichment and the generation of glycolytic intermediates. Core genes participating in glycolysis and the TCA cycle, namely *GPI*, *PFK*, *FBA1*, *TPI*, *PK*, *PDHC*, *ACO*, *SDH* and *MDH*, were synchronously upregulated at 13:00 to enhance energy supply; their expression declined in the early morning and late afternoon to maintain basal metabolic demands. Key genes of the shikimate–phenylpropanoid pathway (*CM*, *PAL*, *C4H*) were significantly upregulated at 13:00, providing precursors for flavonoid biosynthesis. Similarly, flavonoid biosynthetic genes (*CHS*, *CHI*, *FLS*, *ANA*) peaked at 13:00, promoting biosynthesis of quercetin, anthocyanins and catechins, whereas their transcript levels declined at 7:00 and 18:00. Overall, tea shoots coordinately upregulated the genes for carbon metabolism and flavonoid biosynthesis at 13:00, but prioritized starch degradation and basal metabolism at 7:00 and 18:00, forming a temporal regulatory pattern for carbon allocation and quality-related metabolite accumulation.

**Figure 7:**
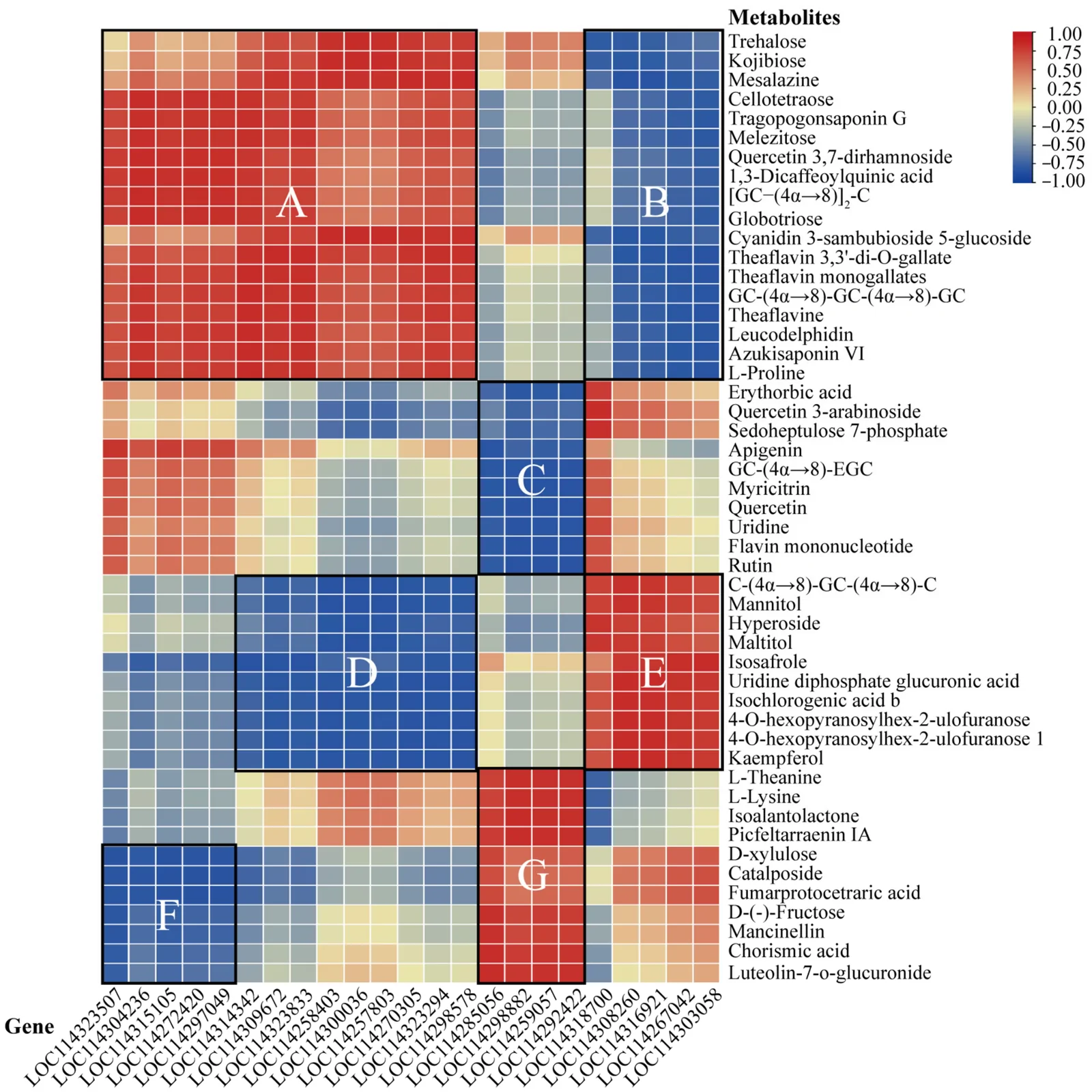
Correlation analysis between selected DAMs and DEGs. The heatmap displays Pearson correlation coefficients for 23 DEGs and 49 DAMs. Red denotes a positive correlation, blue indicates a negative correlation, and deeper color intensity represents a stronger correlation. Statistical significance was determined using Pearson’s correlation test, and only correlations with a *p*-value < 0.05 are shown. Gene names for each locus symbol are listed in Table S5.

We further analyzed correlations between 23 DEGs and 49 DAMs (Figure 7, Table S5). Metabolites in Module A, including oligosaccharides, flavonoids, catechins and theaflavins, exhibited strong positive correlations between their abundance and the expression of corresponding genes. Module E predominantly consisted of gallocatechin-(α-8)-epigallocatechin, quercetin and kaempferol, while Module G comprised amino acids, terpenoids, saccharides, iridoid glycosides and flavonoids. The accumulation levels of metabolites from Modules E and G were also strongly positively correlated with the transcript abundance of their matched genes. In contrast to Modules A/E/G, metabolites in Modules B/C/D/F showed negative correlations with the expression of corresponding genes. Therefore, across these daytime sampling points, each metabolite module was linked to specific genes; temporal variations in gene expression led to differential accumulation of primary and secondary metabolites, uncovering a finely regulated daytime carbon allocation trade-off in tea plants.

### 2.5. Reverse-Transcription Quantitative PCR (RT-qPCR) Validation of Key DEGs Expression Levels

**Figure 8:**
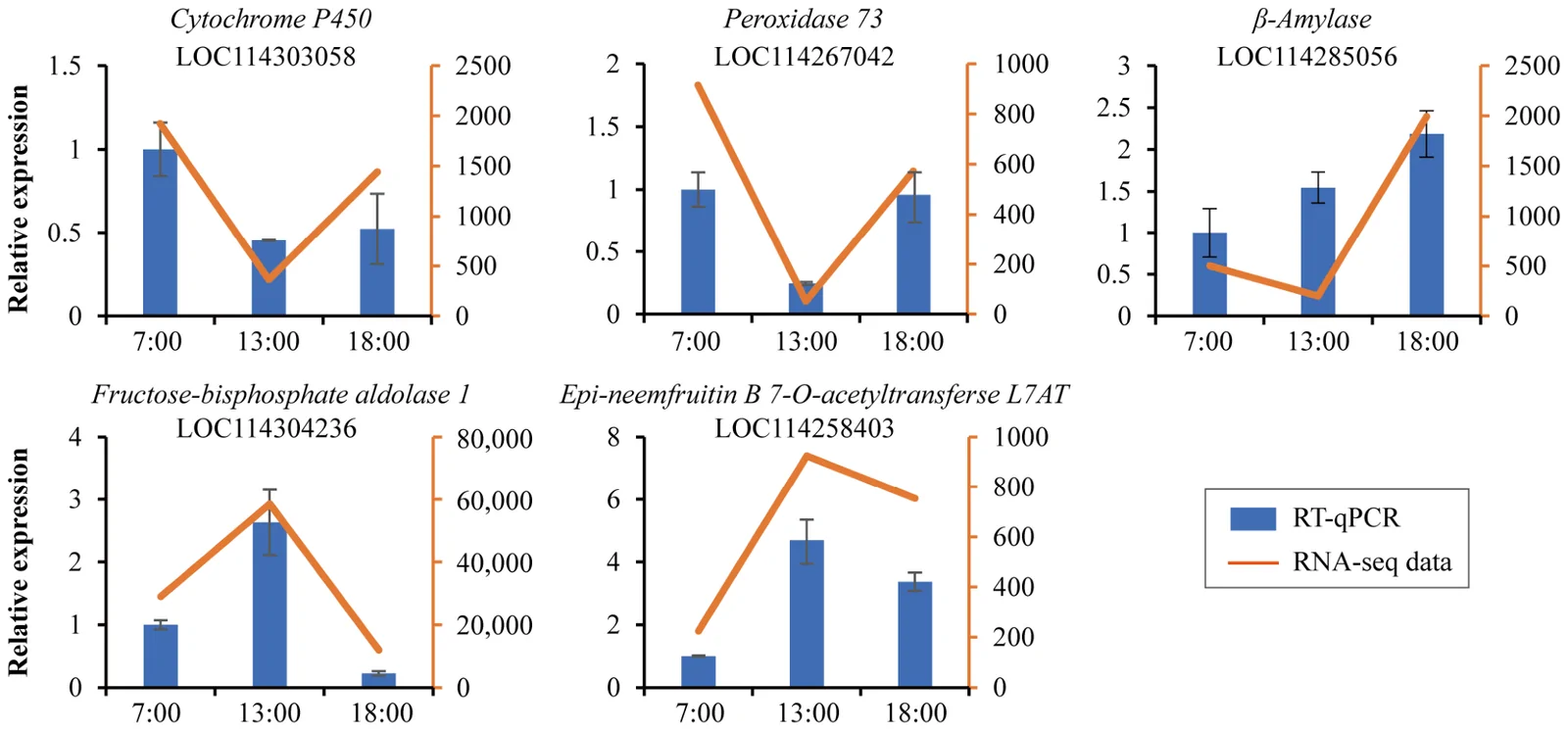
RT-qPCR validation of five DEGs. The bar chart presents RT-qPCR results expressed as mean ± SD, with relative expression normalized to the 7:00 group. The line graph shows the average gene expression levels (*n* = 3) derived from RNA-seq data.

To verify the reliability of RNA-seq data, five DEGs were screened for RT-qPCR analysis of their expression in tea shoots at three daytime sampling time points (Figure 8). *CYP72A219* (LOC114303058) and *peroxidase 73* (LOC114267042) exhibited identical time-of-day-dependent expression profiles (lowest at 13:00, highest at 7:00). Both were down-regulated at 13:00, suggesting that midday light/heat stress could transiently suppress antioxidant and terpenoid modification pathways in tea leaves. In contrast, *fructose-bisphosphate aldolase 1* (*FBA1*, LOC114304236) and *epi-neemfruitin B 7-O-acetyltransferase L7AT* (LOC114258403) peaked at 13:00. As a key Calvin cycle enzyme, FBA1 promotes midday photosynthetic carbon fixation to supply carbon skeletons for secondary metabolism. Meanwhile, high *L7AT* expression indicates active limonoid acetylation at noon. Chloroplast *BAM3* (LOC114285056) peaked at 18:00, indicating enhanced starch degradation in the late afternoon to support the synthesis of flavonoids, terpenoids and other quality-related metabolites. Overall, all five validated genes showed temporal specificity of expression patterns, consistent with RNA-seq results.

## 3. Discussion

### 3.1. Time-of-Day-Dependent Metabolic Trade-Offs and Quality Divergence of Tea Shoots

Over the course of the daytime period, environmental factors (light, temperature, humidity) in the XY10 tea plantation fluctuate significantly, directly influencing photosynthesis and quality formation. We found that leaf physiological indices (starch, soluble sugars, chlorophyll, polyphenols, RWC) and metabolomic profiles underwent obvious time-of-day-dependent changes across sampling times (Figure 3). Leaf maturity and sampling time significantly affected these traits. Starch content was negatively correlated to leaf tenderness; the second fully expanded leaf showed stronger diurnal starch accumulation than buds and the first leaf, consistent with the conservative anatomy of *Camellia* plants [26]. In tea leaves, starch granules displayed distinct accumulation patterns across mesophyll tissues and midrib vascular bundles over the course of the day (Figure 2, Figure 3 and Figure S1), and starch content rose steadily from morning to evening, peaking at 18:00, and was hydrolyzed to soluble sugars overnight, reaching its lowest at 7:00—a turnover pattern consistent with *Arabidopsis*, maize and citrus [22,23,27,28]. Two other key physiological indices showed regular time-of-day-dependent variations correlated with light intensity (Figure 3). Chlorophyll content rose with light from 7:00 to 13:00 and peaked at midday. RWC exhibited the opposite trend: it was highest at 7:00 owing to low nocturnal transpiration, declined to a minimum at midday with intensified transpiration, and gradually recovered after 18:00. Collectively, these physiological shifts likely arise from a dynamic balance among photosynthetic carbon fixation, transpiratory water loss, and high-light stress adaptation across the daytime period.

Starch acts as a major temporary carbon pool and regulates tea physiology, metabolism and quality. It is degraded to soluble sugars through glycolysis and the TCA cycle (Figure 6), which facilitate the synthesis of flavor metabolites such as polyphenols, caffeine and theanine [24,29]. Its glycolytic intermediates provide precursors for theanine, caffeine, polyphenols and flavonoids [30]. Postharvest handling increases amylase activity and starch degradation, improving tea sensory quality [31]. Starch correlates negatively with most catechins but positively with EGCG and CG [32]. We also found positive mutual correlations among starch, soluble sugars and theanine, and among theaflavins, theaflavin monogallates and catechin oligomers (Figure 5B). Accordingly, time-of-day-dependent changes in starch modulate secondary metabolite accumulation and strongly influence the flavor of processed tea.

Integrative metabolomic and transcriptomic profiling of tea cultivar XY10 revealed pronounced time-of-day-dependent variation of 525 DAMs (Figure 4, Table S1), indicating active environmental adaptation and metabolic reprogramming. Flavor-related metabolites (theanine, catechin oligomers, flavonols) exhibited clear time-of-day-dependent changes and determined tea flavor (Table S1). Catechins contribute to astringency and bitterness, while theanine produces umami taste [14]. Flavonoids, mainly flavonol glycosides and proanthocyanidins, are structurally diverse across tea varieties [4]. Tea polyphenols, theanine, catechins and flavonoids in XY10 exhibited distinct time-of-day-dependent changes, and their profiles resemble the characteristic metabolites of other Chinese green teas [4,10,14]. The metabolic patterns in tea shoots are governed by precise trade-offs in carbon allocation. At 13:00, active photosynthesis and carbon fixation boost primary metabolism, producing carbohydrates, starch, and theanine that supply carbon skeletons and energy for growth; while secondary metabolic pathways are activated in the afternoon and evening, causing abundant accumulation of polyphenols, flavonoids and theaflavins. The strong negative correlation between primary and secondary metabolism (Figure 5 and Figure 7) verified this daytime carbon trade-off, a conserved feature among *Camellia* species modulated by environmental fluctuations and genetic factors [16,27].

Furthermore, we detected clear differences in metabolite patterns between this study and the seasonal research of Gong et al. (2020) [16]. They reported higher theanine content in spring versus summer and autumn, with catechins reaching maximum levels in summer. By contrast, our results showed catechin polymers peaked at 7:00 and gradually declined, while theanine accumulated continuously during daytime (Table 1). Such discrepancies imply tea plants use different carbon allocation strategies for time-of-day-dependent responses and seasonal adaptation. Moreover, caffeine displayed distinct time-of-day-dependent fluctuations but no significant seasonal or phylogenetic variation [4,16], which reflects the complexity of temporal metabolic regulation in tea plants.

Based on our findings, we propose that fresh XY10 shoots harvested in the early morning are suitable for making high-astringency green tea, while evening-harvested shoots contain higher levels of amino acids and soluble sugars and are ideal for producing fresh-mellow green tea, lightly fermented tea and black tea.

### 3.2. Transcriptional Regulation of Time-of-Day-Dependent Metabolic Variation in Tea Shoots

Most secondary metabolites in leaves are primarily synthesized in daytime under the control of light, carbon supply and time-of-day-associated variation [28]. Light induces PAL, CHS and F3H to activate the phenylpropanoid pathway and increase polyphenols; high light and UV further promote flavonoid, theanine and terpenoid accumulation [19,33,34]. Histone modifications also regulate light-dependent secondary metabolism [21].

In this study, a total of 19,767 DEGs were identified in tea shoots at the three sampling time points (Table S3). Light-responsive TFs (Figure 6; Table 2) and other functional genes mediate the time-of-day-dependent shift between primary and secondary metabolism. *CRD1* (LOC114272420), *FBA1* (LOC114304236) and the *P-loop containing nucleoside triphosphate hydrolase* (LOC114255883) control carbon fixation and energy metabolism. Genes peaking at 13:00 maintain primary metabolism, whereas elevated *BAM3* (LOC114285056) at 18:00 promotes starch breakdown and produces metabolic precursors. These primary metabolic reactions support subsequent secondary metabolism. Genes expressed mainly at 7:00 and 18:00, such as *peroxidases* and *CYP72A219* (LOC114303058), regulate antioxidant defense and the biosynthesis of polyphenols, flavonoids, terpenoids and alkaloids, together with chloroplastic *polyphenol oxidase* (LOC114323507), *3-hydroxy-3-methylglutaryl coenzyme A reductase 2-B* (LOC114314342) and *tropinone reductase* (LOC114318700). *ABA2* (LOC114297049) and *NCED4* (LOC114323833) further modulate central carbon metabolism to coordinate the two metabolic pathways. However, gene expression does not always correlate with metabolite levels. For instance, nine DEGs in the caffeine pathway caused no obvious change in caffeine content [4], suggesting the involvement of post-transcriptional regulation.

Importantly, several major TF families were enriched at different time points (Table 2), with MYB, AP2/ERF, WRKY, NAC and GRAS being predominant. Previous studies have demonstrated that MYB, bHLH, WRKY, AP2/ERF, and TCP transcription factors, along with UDP-glycosyltransferases (UGTs) and O-methyltransferases (OMTs), co-regulate flavonoid biosynthesis and modification [35]. Consistent with these findings, our results revealed that MYB was significantly enriched at 13:00 to modulate flavonoid and polyphenol biosynthesis. AP2/ERF exhibited peak expression at 18:00, participating in caffeine biosynthesis and stress adaptation. WRKY, NAC, and GRAS positively modulate flavonoid and theanine accumulation. Moreover, bHLH interacts with MYB to form the MBW complex, which cooperatively governs flavonoid metabolism. Taken together, these light-responsive TFs display distinct time-of-day-dependent expression patterns, underlying the light-dependent transcriptional regulation of metabolite levels in tea plants.

Time-of-day-dependent metabolic shifts drive quality differences in tea shoots harvested at different times. Tea shoots sampled at 7:00 have high chlorophyll and low soluble sugars, favoring catechin synthesis. At 13:00, the *BAHD acyltransferase* gene (LOC114258403) promotes theaflavin production, whereas high polyphenol oxidase expression reduces polyphenol levels. At 18:00, elevated *BAM3*, amino acids and soluble sugars make evening tea shoots ideal for lightly fermented tea [36]. Evening tea shoots also contain the highest gallocatechin polymers, which are readily oxidized during black tea withering and fermentation [37]. Meanwhile, evening tea shoots contain abundant theanine for umami, with hydrolyzed sugars boosting sweetness and amino acid precursors enhancing aroma [38]. Additionally, the accumulation of phenylpropanoid, amino acid, and caffeine metabolism exhibits obvious tissue specificity [39]. Therefore, studying buds and leaves separately will deepen our understanding of the transcriptional regulation governing time-of-day-dependent metabolic changes in tea plants.

## 4. Materials and Methods

### 4.1. Plant Materials and Sampling

Three-year-old *Camellia sinensis* cv. Xinyang 10 was used as experimental material. As an artificially bred cold-tolerant cultivar screened from XYMJ landrace populations via single-plant selection [40], XY10 is a superior cultivar widely applied for XYMJ tea production. The tea plants were grown in a tea plantation at Bailong pool (113°54′47″ E, 32°00′02″ N; altitude: 600 m), Shihe District, Xinyang City, Henan Province, China, under conventional field management.

Tea plant shoots (one bud and one leaf) were randomly harvested from 150 tea plants with uniform size and growth status for physiological, transcriptomic and metabolomic analyses. The plants were randomly divided into three groups for biological replicates (16–17 individual plants per biological replicate). For each biological replicate, shoots collected from the corresponding group of plants were pooled as one composite sample. Sampling was performed at 7:00, 13:00, and 18:00 on a sunny day during the Grain Rain period (around 20 April 2025), with three independent biological replicates for each time point using different plants across time points. After collection, all samples were snap-frozen in liquid nitrogen and stored at −80 °C for subsequent RNA isolation, metabolite extraction, and physicochemical analysis. Light intensity was measured using a pocket-sized illuminance meter (LX20, Sanwa Electric Instrument Co., Ltd., Tokyo, Japan), while ambient air temperature and RH in the tea canopy were synchronously recorded.

### 4.2. Light Microscopy and Iodine Staining of Tissue Sections

Fresh tissue segments (2 mm × 4 mm) were excised from the central regions of buds, first leaves, and second leaves of XY10 tea plants. The samples were fixed in formalin-acetic acid-alcohol fixative (FAA) for 24 h, and softened for 3 d in a dehydration box. Afterwards, the tissues were subjected to gradient dehydration with distilled water and ethanol, cleared with xylene, infiltrated with paraffin, and embedded in paraffin blocks. The embedded blocks were sliced into 3 μm continuous sections, followed by flattening, dewaxing, and staining with either Safranin-Fast green staining or iodine staining, as described in our recent work [23]. Permanent slides were prepared for microscopic observation. The tissue microstructure and starch accumulation characteristics were visualized and photographed under a light microscope.

### 4.3. Determinations of Main Chemical Compositions

RWC in tea tissue samples was determined in accordance with China National Standard GB/T 8304-2013. Starch content was determined using a commercial assay kit (AKSU015C, Boxbio, Beijing, China) following the manufacturer’s standard protocols. Soluble sugar content was measured using a commercial assay kit (AKPL008C, Boxbio, Beijing, China) following the manufacturer’s instructions. Chlorophyll a, chlorophyll b and total chlorophyll contents of leaves were extracted with 95% ethanol, quantified spectrophotometrically, and calculated via ethanol-based formulas [41]. Total tea polyphenols were extracted as previously described [24] and quantified using the modified Folin–Ciocalteu method specified by ISO 14502-1 [42]. Gallic acid was used as the standard for calibration. The absorbance of each sample was measured at 765 nm with ultrapure water serving as the blank control.

### 4.4. Total RNA Extraction

Total RNA was extracted using TRIzol reagent (Promega, Beijing, China) according to the manufacturer’s instructions, with three biological replicates for each group. RNA purity was assessed using a NanoDrop One/One^c^ Spectrophotometer. RNA integrity was evaluated via a QSEP400 automated capillary electrophoresis system, and accurate RNA quantification was assayed using a Qubit^®^ 4.0 fluorometer with a Qubit™ RNA HS Assay Kit (Invitrogen, Thermo Fisher Scientific, Carlsbad, CA, USA). Qualified RNA samples were used for RNA-seq and RT-qPCR validation.

### 4.5. RNA-Seq and Bioinformatics Analysis

Total RNA extracted from XY10 one-bud-one-leaf shoots collected at different intra-day time points was used to construct three independent RNA-seq libraries. Library sequencing was performed on the Illumina NovaSeq 6000 platform by Kindstar Sequenon Biotechnology Co., Ltd. (Wuhan, China). Briefly, mRNA was enriched using oligo(dT) magnetic beads, and first-strand and second-strand cDNA were synthesized via the M-MuLV reverse transcriptase system. After purification and adapter ligation, qualified cDNA libraries were subjected to high-throughput sequencing. The raw sequencing reads were filtered to obtain clean reads, which were then aligned to the reference genome of *C. sinensis* var. *sinensis* (AHAU_CSS_1, [43]) using HISAT2 software (v.2.2.1). Gene expression levels were normalized and calculated as fragments per kilobase of transcript per million mapped reads (FPKM). DEGs were defined with an FPKM value > 50.

Three pairwise differential comparison groups were set: 18:00 vs. 7:00, 18:00 vs. 13:00, and 13:00 vs. 7:00. DEGs were screened using the DESeq2 R package with the threshold of |log_2_FC| ≥ 1 and adjusted *p*-value < 0.05. The Benjamini–Hochberg false discovery rate (FDR) correction was applied to correct for multiple testing. TBtools software (v.2.390) [44] was used to generate heatmaps, Venn diagrams, and correlation analyses for key tea quality-related genes and metabolites.

To explore the potential biological functions of DEGs in tea plants, Gene Ontology (GO) functional enrichment analysis was performed using hypergeometric distribution tests. KEGG pathway enrichment analysis was conducted using the clusterProfiler (v3.4.4) package. A threshold of *p* < 0.05 was adopted to identify significantly enriched terms in both GO and KEGG analyses.

### 4.6. Metabolome Profiling and Bioinformatics Analysis

XY10 tissue samples were freeze-dried and ground into fine powder using a Retsch MM 400 Mixer Mill (Haan, Germany) at 30 Hz for 1.5 min. Briefly, 30 mg of the powdered sample was homogenized with 70% aqueous methanol for metabolite extraction. The mixture was vortexed for 30 s every 30 min six times, followed by centrifugation at 12,000 rpm for 3 min. The supernatant was filtered through a 0.22 μm organic membrane and subjected to untargeted metabolomic analysis [10]. Metabolite detection was performed on an ACQUITY UPLC system (Waters Corp., Milford, MA, USA) equipped with an Acquity HSS T3 column (1.8 μm, 100 mm × 2.1 mm) and coupled with a Xevo G2-XS QTof mass spectrometer. The column temperature was maintained at 40 °C, and the flow rate was set to 0.40 mL/min with a 4 μL injection volume. QC samples were prepared by mixing equal volumes of all sample extracts to monitor system stability and data reproducibility. The mobile phase consisted of 0.1% formic acid aqueous solution (phase A) and acetonitrile (phase B). Gradient elution was performed as follows: initial 95% A/5% B; 0–5 min to 35% A/65% B, 5–6 min, linear gradient to 1% A/99% B for 1.5 min; rapid return to 95% A/5% B within 0.1 min, followed by 2.4 min of re-equilibration.

Mass spectra were acquired in information-dependent acquisition (IDA) mode using Analyst TF 1.7.1 software (SCIEX, Concord, ON, Canada). Ion source parameters were set as follows: GAS1, 50 psi, GAS2, 60 psi, CUR, 35 psi, 550 °C, DP, ±80 V, ISVF, 5500 V/−4500 V. The product ion scanning range was 50–1250 Da with an accumulation time of 40 ms, collision energy of ±30 V (15 V energy spread), unit resolution, intensity threshold of 100 cps, 4 Da isotope exclusion, 50 mDa mass tolerance, and 12 candidate ions per cycle.

Raw mass spectra were converted to mzML using ProteoWizard software (v. 3.0) [45]. Peak picking, alignment, and retention time correction were performed via XCMS software [46]. Features with coefficient of variation (CV) > 0.5 across QC samples were removed to reduce false-positive metabolite features. Peaks with a missing value rate exceeding 50% were discarded, and remaining missing values were imputed using the K-nearest neighbor (KNN) method or 1/5 of the minimum peak value. All peak areas were normalized via support vector regression (SVR). Metabolites were annotated against MetDNA and KEGG Compound databases (http://www.kegg.jp/kegg/compound/, accessed on 6 November 2025). Only metabolites with an identification score ≥ 0.5 were retained. Annotated metabolites were mapped to the KEGG pathway database for functional pathway interpretation.

PCA was performed using the prcomp function in R software (v.4.4.1) to evaluate sample clustering and repeatability. HCA, heat maps and Pearson correlation coefficient (PCC) were generated using the ComplexHeatmap package (v.2.28.0) in R; for HCA, normalized signal intensities of metabolites were visualized as a color spectrum. For two-group analysis, orthogonal partial least squares discriminant analysis (OPLS-DA) was performed using the R package MetaboAnalystR (v.4.0) after log_2_ transformation and mean centering of the data; a 200-time permutation test was performed to prevent model overfitting. DAMs were identified with the thresholds of VIP > 1 and |log_2_FC| ≥ 1.0. TBtools software was used to construct the correlation network between key DAMs and DEGs [44]. For correlation analysis, Pearson correlation coefficients were calculated between the normalized abundances of DAMs and the FPKM values of DEGs. Statistical significance of each correlation was determined using a two-sided Pearson correlation test, and only correlations with a *p*-value < 0.05 were considered statistically significant and displayed in the heatmap.

### 4.7. RT-qPCR Validation

Gene-specific primers were designed using Primer Premier 5.0 software and commercially synthesized at the Biomed Cooperation (Beijing, China) (Table S6). *β-Actin* was used as the internal reference gene. Total RNA was extracted using the TRIzol method and reverse-transcribed into cDNA using the 5×All-in-One MasterMix kit (Applied Biological Materials Inc., Richmond, BC, Canada). The reaction system of RT-qPCR was made using the kit of Hieff^®^ qPCR SYBR Green Master Mix (YEASEN, Shanghai, China) and performed on a StepOnePlus Real-Time PCR System (Thermo Fisher Scientific, Carlsbad, CA, USA). Relative expression levels were calculated using the 2^−ΔΔ*CT*^ method [47]. All experiments were performed in three biological replicates, and data were presented as the mean ± standard deviation (SD).

### 4.8. Statistical Analysis

Data were statistically analyzed using SPSS software (v.26.0, SPSS Inc., Chicago, IL, USA). One-way analysis of variance (ANOVA) was performed, followed by Duncan’s multiple range test to evaluate significant differences among groups (*p* < 0.05).

## 5. Conclusions

**Figure 9:**
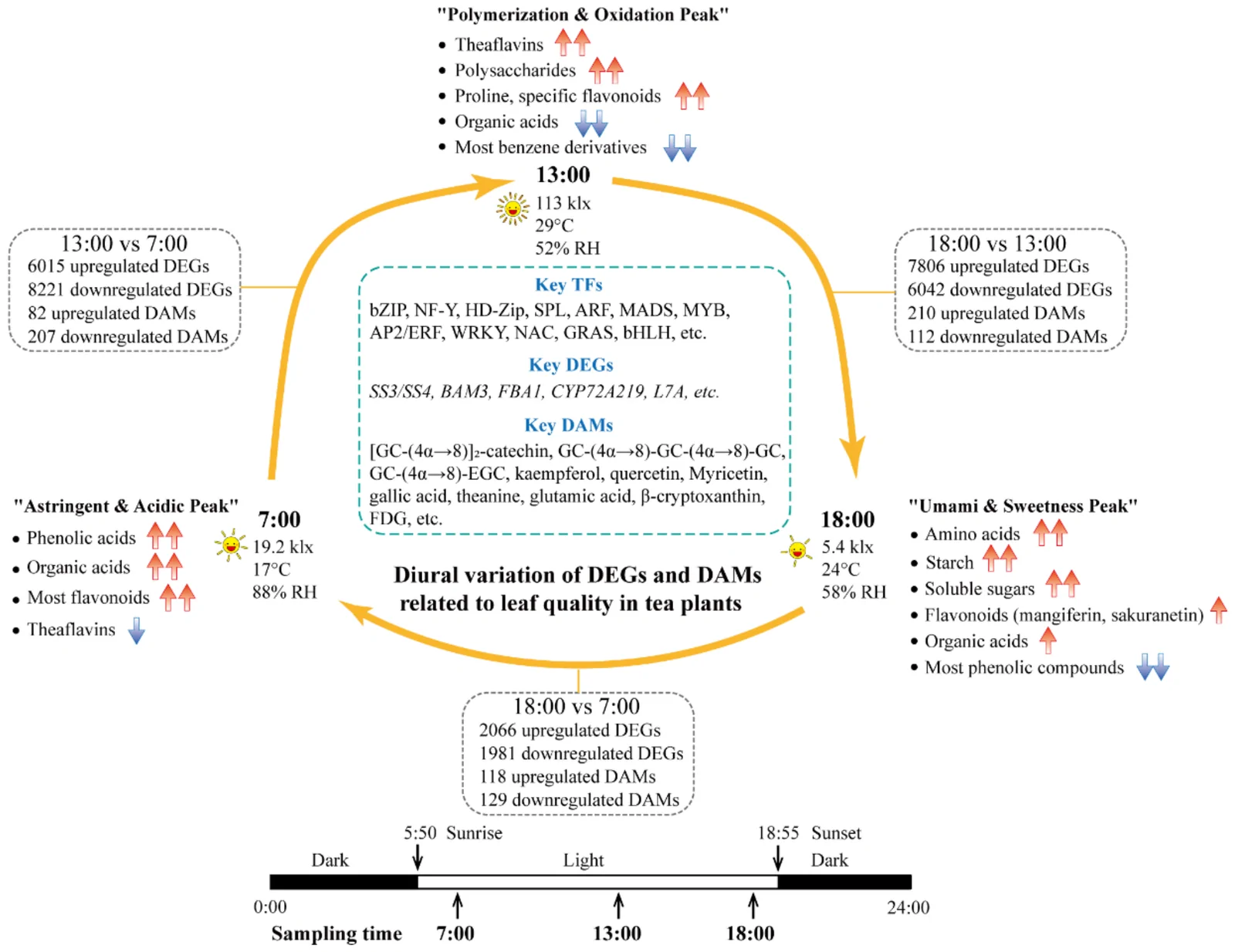
Summary of daytime changes in the transcriptome and metabolome in tea leaves and their effects on leaf quality. Multi-omics integration uncovered time-dependent transcriptional reprogramming controlling the biosynthesis of taste and aroma compounds, which jointly determines the time-of-day-dependent variation of fresh tea leaf quality. Upward arrows represent increased metabolite accumulation, while downward arrows represent decreased metabolite accumulation.

This study reports the first integrated transcriptomic and metabolomic analysis of time-of-day-associated changes in major metabolites of XYMJ tea plants. The results show that primary metabolism peaks at noon and secondary metabolism in the evening, with time-point-specific genes regulating core pathways and key flavor compounds (Figure 9). Leaves harvested at different times differ in metabolic characteristics and processing suitability for green and black tea. Time-dependent accumulation of bioactive compounds highlights the benefits of timely harvesting for improving the yield, purity and extraction efficiency of target components. These findings advance tea time-of-day-dependent metabolism theory and underpin optimized harvesting, graded processing and precise quality control. However, sampling in this study was confined to the Grain Rain period, so the stability of time-of-day-associated metabolic patterns across summer and autumn still needs confirmation. This study only sampled three daytime time-points. Further well-resolved 24–48 h time-series sampling combined with rhythmic statistical modelling is required to disentangle bona fide endogenous circadian rhythms from direct environment-driven time-of-day-associated responses. Follow-up research will verify the functions of core genes and TFs, and evaluate the quality of processed tea harvested at different time points of the day, thereby improving the theoretical framework for time-of-day-dependent metabolism and quality regulation in tea plants.

## Figures and Tables

**Table 1:** Changes in the relative abundance of 54 key secondary metabolites in tea samples at three harvesting times.

Compounds	Related to Tea Quality	7:00	13:00	18:00
Alcohol and amines				
β-Cryptoxanthin	Liquor color	3195.93 ± 113.27 ^b^	2111.77 ± 403.56 ^c^	4613.21 ± 1153.73 ^a^
FDG	Aroma precursor	9520.09 ± 1070.77 ^b^	9680.47 ± 664.13 ^b^	28,331.06 ± 748.68 ^a^
HMPP-G	Sweetness, mellow and returning sweetness; roast fragrance	20,351.02 ± 1082.16 ^b^	6741.99 ± 1169.38 ^c^	39,496.97 ± 1220.57 ^a^
Amino acids and derivatives				
D-Pyroglutamic acid	Sweetness, fresh-briskness	11,865.55 ± 942.42 ^c^	16,630.34 ± 1416.57 ^b^	29,662.69 ± 1354.08 ^a^
Glutathione (reduced)	Liquor color, browning	72,729.08 ± 9352.52 ^a^	25,799.94 ± 2349.30 ^b^	37,336.10 ± 5844.75 ^b^
L-Proline	Sweetness, nutty roasted aroma precursor	9272.08 ± 630.81 ^b^	24,442.92 ± 2575.49 ^a^	11,839.04 ± 2005.83 ^b^
L-Lysine	roast aroma precursor	18,995.20 ± 571.82 ^c^	28,172.72 ± 100.57 ^b^	48,063.03 ± 4169.92 ^a^
L-Theanine	Fresh-briskness, sweet and mellow, aroma precursor	404,497.46 ± 7292.80 ^c^	605,747.18 ± 20,876.40 ^b^	1,053,371.28 ± 75,292.25 ^a^
Benzene and derivatives				
2-Hydroxy-3,4-dimethoxybenzoic acid	Slight sourness, slight astringency	19,116.79 ± 6625.56 ^a^	10,510.77 ± 2287.31 ^b^	7425.37 ± 1976.97 ^b^
4,5-Dihydroxyphthalic acid	Slight sourness, slight astringency	199,047.98 ± 40,726.57 ^a^	79,168.67 ± 12,997.91 ^b^	104,997.17 ± 12,644.68 ^b^
6-MGDF	Slight astringency, Liquor mellowness	129,674.02 ± 9070.86 ^a^	44,684.77 ± 4851.63 ^b^	41,173.61 ± 1180.97 ^b^
FDF-Gly	Fresh floral sweetness, soft mellowness	552,552.60 ± 47,175.17 ^b^	315,835.63 ± 10,663.74 ^c^	716,745.30 ± 32,842.02 ^a^
HDPFAA	Aged aroma, sweet mellowness	6695.93 ± 1596.38 ^b^	6360.12 ± 1661.34 ^b^	19,730.33 ± 1235.11 ^a^
Naringenin 7-o-glucoside	Refreshing bitterness, aroma base	21,092.36 ± 1089.42 ^a^	13,523.34 ± 1483.04 ^b^	2203.13 ± 1440.56 ^c^
Procyanidin Trimer-Vanillyl 1	Strong astringency, thick viscous texture	38,618.48 ± 967.21 ^b^	191,899.96 ± 14,579.16 ^a^	42,348.99 ± 7344.51 ^b^
Flavonoids				
Cyanidin 3-arabinoside cation	Purple-orange redness	17,292.07 ± 125.01 ^a^	7656.32 ± 783.81 ^c^	11,236.02 ± 2252.80 ^b^
Cyanidin 3-sambubioside 5-glucoside	Red brightness	1643.37 ± 340.83 ^b^	3968.41 ± 1595.64 ^a^	3402.81 ± 762.34 ^a^
[GC-(4α→8)]_2_-catechin	Astringency, mellow thickness	1033.67 ± 74.16 ^b^	3190.63 ± 669.97 ^a^	824.79 ± 161.17 ^b^
GC-(4α→8)-GC-(4α→8)-GC	Astringency, mellow thickness	16,137.83 ± 229.89 ^c^	32,893.55 ± 5744.52 ^a^	19,409.97 ± 2305.61 ^b^
GC-(4α→8)-EGC	Astringency, mellow thickness	30,593.59 ± 2435.66 ^a^	29,955.21 ± 2161.46 ^a^	14,538.46 ± 4053.80 ^b^
Hyperoside	Briskness, mellowness, anti-browning	502,936.59 ± 22,940.28 ^a^	228,641.04 ± 5822.01 ^b^	226,301.21 ± 19,218.90 ^b^
K3G-3″-Rha	Sweetness	175,097.70 ± 4049.66 ^b^	192,857.06 ± 9521.37 ^a^	65,374.13 ± 1208.05 ^c^
Kaempferol	Briskness, fresh sweetness, after-sweetness	6558.69 ± 1112.30 ^a^	2974.21 ± 266.38 ^c^	4299.71 ± 536.07 ^b^
Kaempferol 3-galactoside-7-rhamnoside	Sweet freshness, briskness, antioxidation	294,792.57 ± 69,173.41 ^a^	331,812.71 ± 46,815.22 ^a^	58,025.19 ± 32,941.88 ^b^
Maclurin	Taste, flavor	175,781.40 ± 63,251.10 ^a^	74,127.12 ± 4733.59 ^b^	186,461.87 ± 753.42 ^a^
Mangiferin	Briskness, fresh sweetness, after-sweetness	19,023.07 ± 1978.62 ^b^	13,507.64 ± 3117.78 ^c^	33,948.32 ± 7690.58 ^a^
Morin	Flavor, liquor color	100,096.35 ± 3628.21 ^b^	211,910.77 ± 8714.41 ^a^	133,615.55 ± 25,764.21 ^b^
Myricitrin	Taste, liquor color, aroma	29,105.62 ± 2915.23 ^a^	27,142.86 ± 1251.90 ^a^	9405.89 ± 3881.27 ^b^
Naringenin-7-O-D-Glucuronide	Flavor, liquor color	85,628.07 ± 2758.64 ^c^	408,471.10 ± 173,200.90 ^b^	837,622.74 ± 345,152.96 ^a^
Quercetin	Taste, flavor	76,767.80 ± 2713.31 ^a^	71,793.93 ± 2391.51 ^a^	30,697.42 ± 1222.93 ^b^
Quercetagetin	Elegant sweet aroma, briskness	32,223.44 ± 2327.65 ^a^	15,197.68 ± 1946.82 ^c^	21,033.16 ± 1813.94 ^b^
Rutin	Briskness, mellowness, anti-browning	1,078,985.79 ± 77,536.19 ^a^	917,364.87 ± 61,684.30 ^b^	183,366.26 ± 51,560.12 ^c^
Sakuranetin	Floral aroma, sweet aroma	442,987.45 ± 9321.10 ^b^	235,978.28 ± 66,372.71 ^c^	515,472.68 ± 15,900.33 ^a^
Organic acids				
4-(β-D-glucosyloxy)benzoic acid	Aroma precursor	84,535.02 ± 4128.23 ^a^	33,834.39 ± 1830.98 ^b^	25,977.51 ± 3140.26 ^c^
DHAI-Api-Glc	Aroma precursor, antioxidation	1345.99 ± 850.70 ^b^	564.80 ± 53.43 ^c^	3462.96 ± 389.35 ^a^
FC-GlcA	Flavor, liquor color	17,740.14 ± 4503.06 ^b^	5861.18 ± 1209.31 ^c^	41,037.44 ± 2552.73 ^a^
FPD-SA	Flavor, liquor color	10,876.60 ± 758.57 ^b^	9394.07 ± 1182.19 ^b^	21,940.31 ± 2065.11 ^a^
Others				
2,4′-Dihydroxychalcone	Quality, flavor	302.24 ± 86.70 ^c^	2444.78 ± 84.68 ^b^	3974.14 ± 402.60 ^a^
4-O-hexopyranosylhex-2-ulofuranose	Taste quality, aroma	16,823.06 ± 339.99 ^a^	7773.97 ± 1213.83 ^c^	11,207.44 ± 3407.23 ^b^
Cellotetraose	Liquor viscosity, mellowness	733.13 ± 446.47 ^b^	7733.45 ± 1607.53 ^a^	640.46 ± 85.89 ^b^
D-(-)-Fructose	Taste quality	62,475.35 ± 1794.44 ^b^	37,496.35 ± 10,203.74 ^c^	114,343.83 ± 5770.72 ^a^
Mannitol	Taste quality, liquor smoothness	82,629.11 ± 9391.14 ^a^	3114.11 ± 1530.60 ^c^	19,339.87 ± 2902.79 ^b^
Neoxanthin	Xanthophylls, color and antioxidation	1585.66 ± 690.72 ^b^	7849.14 ± 1846.96 ^a^	2941.14 ± 971.35 ^b^
Quercetin 7-O-glucoside	Flavonoid precursor, quality, flavor	9813.58 ± 656.18 ^a^	3099.28 ± 253.62 ^b^	1338.89 ± 724.44 ^c^
Trehalose	Quality flavor, antioxidation	68,916.82 ± 15,849.44 ^b^	142,959.46 ± 12,491.67 ^a^	138,955.39 ± 5539.72 ^a^
Phenolic acids				
1-Caffeoyl-beta-D-glucose	slight bitterness and astringency, antioxidation	6970.84 ± 417.08 ^a^	2954.29 ± 341.00 ^b^	3122.54 ± 1121.82 ^b^
1,3-Dicaffeoylquinic acid	Aroma, taste	76,943.79 ± 24,273.62 ^b^	294,278.70 ± 27,155.69 ^a^	45,351.77 ± 11,786.62 ^c^
3,4-Dihydroxybenzaldehyde	Flavor, aroma	23,320.12 ± 696.45 ^a^	5473.68 ± 335.65 ^c^	15,942.07 ± 466.81 ^b^
Syringic acid	Quality flavor, antioxidation	5678.26 ± 608.48 ^a^	1887.07 ± 365.84 ^c^	3239.21 ± 80.22 ^b^
2-O-Galloyl-D-glucose	Taste quality	40,822.08 ± 2405.87 ^a^	22,085.72 ± 1536.36 ^b^	44,948.28 ± 3531.35 ^a^
Sinapic acid	Flavor, aroma, antioxidation	8060.83 ± 902.13 ^a^	4906.35 ± 1504.43 ^b^	3864.53 ± 1526.88 ^b^
Tannins				
Theaflavin	Flavor, color, antioxidation	26,010.05 ± 1463.75 ^c^	125,729.47 ± 5050.68 ^a^	47,223.94 ± 2855.26 ^b^
Theaflavin 3,3′-di-O-gallate	Flavor, color, antioxidation	6424.46 ± 1378.40 ^c^	31,992.50 ± 4019.79 ^a^	16,205.91 ± 3955.65 ^b^
Theaflavin monogallates	Flavor, color, antioxidation	13,729.25 ± 693.13 ^c^	73,977.16 ± 5102.95 ^a^	28,061.87 ± 2317.40 ^b^

Note: Data represent normalized relative peak areas (semi-quantitative values from untargeted LC-MS metabolomics; no absolute concentrations were determined). All data are expressed as mean ± S.D. (*n* = 3). Different letters in the same row indicate significant differences between mean values (*p* < 0.05, ANOVA, Duncan’s test). 6-MGDF, 3,5,7-trihydroxy-6-methoxy-2-(3,4,5-trihydroxyphenyl)-3,4-dihydro-2H-1-benzopyran-4-one; DHAI-Api-Glc, 3β,6α-Dihydroxy-α-ionol 9-[apiosyl-(1→6)-glucoside]; FC-GlcA, 6-{[4,5-dihydroxy-2-({7-oxo-7H-furo[3,2-g]chromen-9-yl}oxy)oxan-3-yl]oxy}-3,4,5-trihydroxyoxane-2-carboxylic acid; FDF-Gly, 3-((3,4-Dihydroxy-5-(hydroxymethyl)tetrahydrofuran-2-yl)oxy)-2-(3,4-dihydroxyphenyl)-5,7-dihydroxychroman-4-one; FDG, (1R,2R,4S,6R)-2,6-Fenchanediol 2-O-b-D-glucoside; FPD-SA, (3-{8-[1-(2,4-dihydroxyphenyl)-2-hydroxy-3-(3-hydroxyphenyl)propyl]-3,5,7-trihydroxy-3,4-dihydro-2H-1-benzopyran-2-yl}phenyl)oxidanesulfonic acid; [GC-(4α→8)]_2_-catechin, [Gallocatechin-(4α→8)]_2_-catechin; GC-(4α→8)-EGC, Gallocatechin-(4α→8)-epigallocatechin; GC-(4α→8)-GC-(4α→8)-GC, Gallocatechin-(4α→8)-gallocatechin-(4α→8)-gallocatechin. HDPFAA, 2-[3-[3-hydroxy-4-(4-hydroxyphenyl)-2,5-dimethoxyphenyl]-5-oxo-2H-furan-2-yl]acetic acid; HMPP-G, 2-[4-(3-Hydroxypropyl)-2-methoxyphenoxy]-1,3-propanediol 1-glucoside; K3G-3″-Rha, 3″-O-L-Rhamnopyranosylastragalin.

**Table 2:** Comparative analysis of major enriched TFs in three tea tissues sampled at different time points.

TFs	13:00 vs. 07:00	18:00 vs. 13:00	18:00 vs. 07:00	Function
MYB	32.35%	14.4%	14.8%	Core regulation of flavonoid/tea polyphenol biosynthesis (binds to the CHS/LAR promoters)
AP2/ERF	10.0%	38.8%	4.1%	Caffeine biosynthesis; stress response
WRKY	6.5%	1.9%	6.6%	Regulation of flavonoid/caffeine biosynthesis; stress response (binds to W-box)
NAC	6.1%	3.8%	5.7%	Regulation of flavonoid/theanine; senescence response
GRAS	5.0%	2.5%	4.9%	Theanine biosynthesis; hormone signaling (GA/ABA response)
Homeobox/HD-Zip	4.8%	3.3%	2.5%	Organ development; metabolic regionalization
bZIP	4.4%	2.5%	0.8%	Light signal response; regulation of flavonoid/caffeine biosynthesis
TCP	3.5%	2.5%	2.5%	Morphology and secondary metabolite balance of tea plants
General TF	3.3%	0.5%	1.6%	Basal transcription machinery; full-pathway activation
MADS	2.4%	0.8%	4.1%	Spatio-temporal regulation of flower development and metabolism
SPL	2.2%	3.1%	2.5%	Flavonoid biosynthesis; tea plant development (response to miR156)
ARF	1.7%	2.1%	1.6%	Auxin response; spatiotemporal expression of metabolic pathways
Zinc-finger family	1.1%	14.5%	—	Stress response; fine-tuned transcriptional regulation
bHLH	0.9%	3.8%	—	Interacts with MYB to regulate flavonoids (forming the MBW complex)
NF-Y	0.2%	—	—	Light response; synergistic regulation of metabolism
PHD finger	—	0.3%	—	Histone modification; epigenetic regulation, affecting metabolic gene expression via H3K9 methylation
HMGB	—	—	2.5%	Chromatin remodeling; global metabolic activation
Other	15.5%	5.1%	45.9%	—

## Data Availability

The data are contained within the article or Supplementary Materials. The RNA-seq data have been deposited in the NCBI Sequence Read Archive (accession number: PRJNA1387425) and are publicly available. Web link: https://www.ncbi.nlm.nih.gov/sra/PRJNA1387425, accessed on 16 August 2026.

## Supplementary Materials

The following supporting information can be downloaded at: https://www.mdpi.com/article/10.3390/plants15172558/s1, Figure S1. Microstructure and daytime variation of starch accumulation in midrib of leaf2 of XYMJ10. Microscopic sections were prepared and stained with iodine. Scale bar: 50 μm. Figure S2. Overall results of tea transcriptomic data of 9 samples were collected at 7:00, 13:00 and 18:00. (A) Principal component analysis (PCA) of transcriptomic data. (B) Number of DEGs. (C) Venn diagram of DEGs. (D) KEGG analysis of DEGs. Table S1. Major 525 DAMs. Table S2. Full pairwise Pearson correlation-coefficient matrix for key tea metabolites sampled at 7:00, 13:00, and 18:00. Table S3. DEGs identified. Table S4. TFs identified. Table S5. 23 DEGs associated with key metabolites in tea. Table S6. Primers used in this study.
