# Supplementary material for: Integrative Transcriptomic and Metabolomic Analyses of Time-of-Day Variation of Quality-Related Metabolites in Fresh Tea Leaves
Source: Plants (Basel). 2026 Aug 22;15(17):2558. doi: 10.3390/plants15172558 (PMC13567031; doi:10.3390/plants15172558)
Supplement: Supplementary file 1 [file plants-15-02558-s001.zip › Figure S1-S2.pdf]

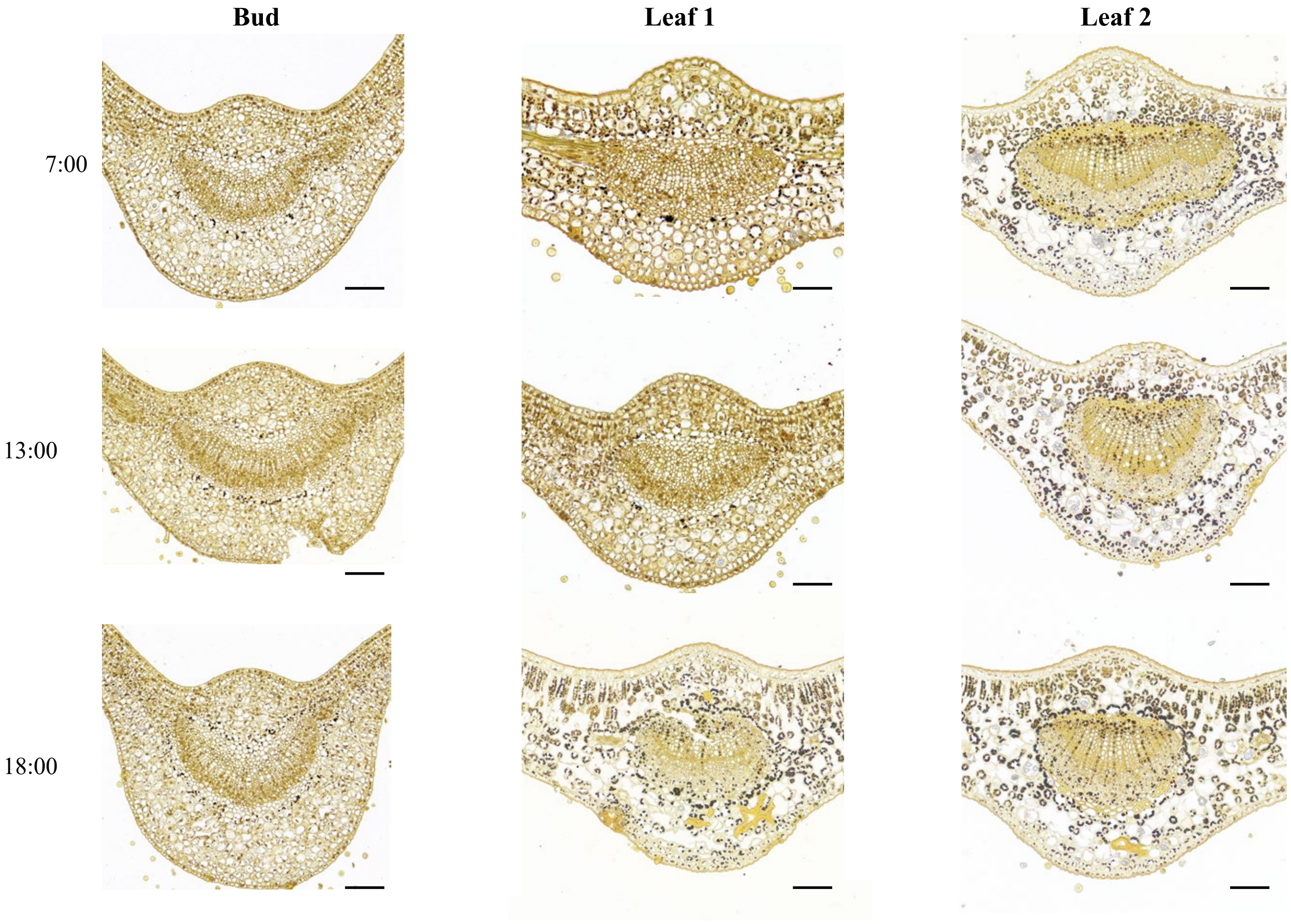

Figure S1. Microstructure and daytime variation of starch accumulation in midrib of leaf2 of XYMJ10 . Microscopic sections were prepared and stained with iodine. Scale bar: 50 μm.

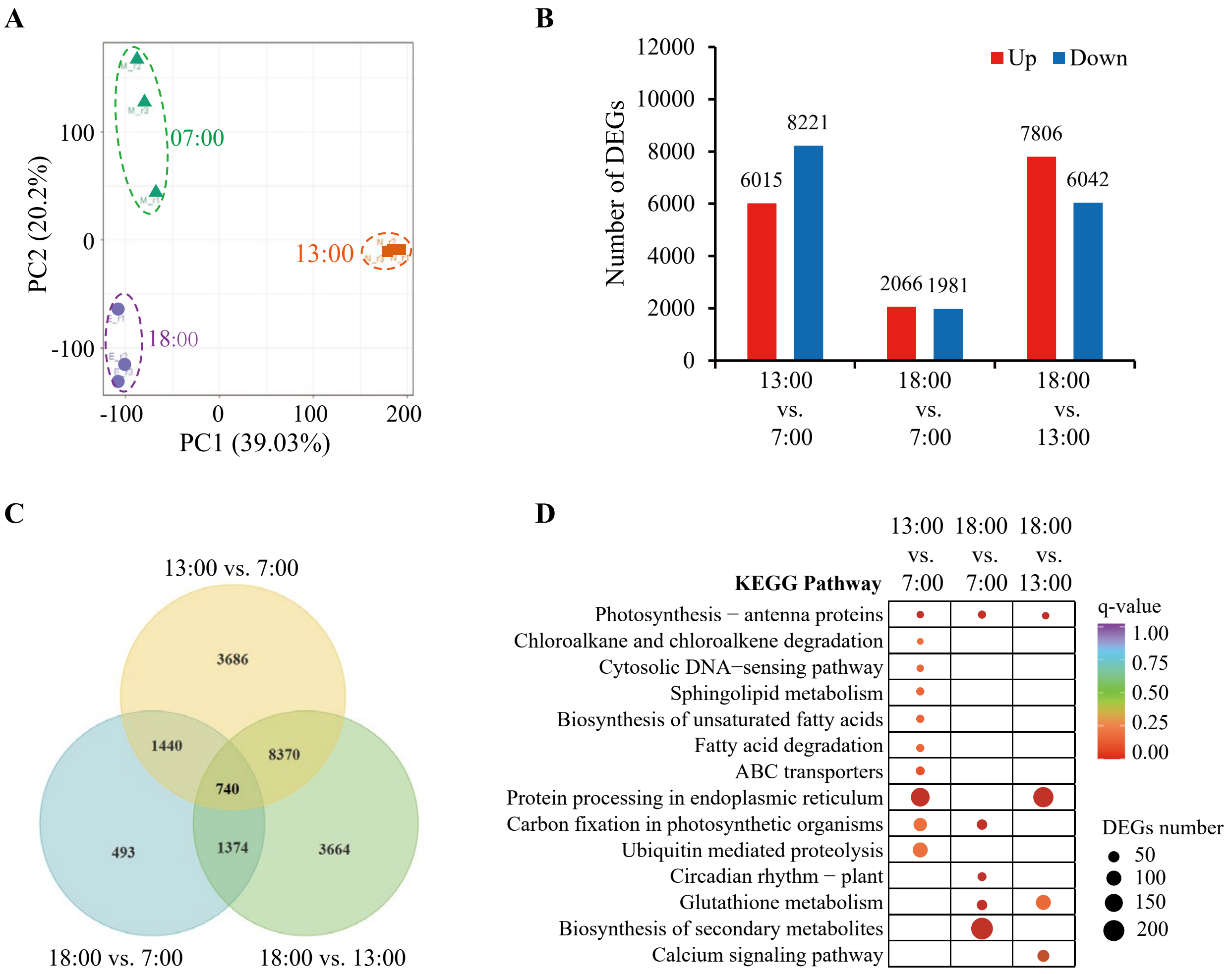

Figure S2. Overall results of tea transcriptomic data of 9 samples were collected at 7:00, 13:00 and 18:00. (A) Principal component analysis (PCA) of transcriptomic data. (B) Number of DEGs. (C) Venn diagram of DEGs. (D) KEGG analysis of DEGs.
